# Effects of adherence to pharmacological secondary prevention after acute myocardial infarction on health care costs – an analysis of real-world data

**DOI:** 10.1186/s12913-020-05946-4

**Published:** 2020-12-20

**Authors:** Florian Kirsch, Christian Becker, Christoph Kurz, Lars Schwettmann, Anja Schramm

**Affiliations:** 1 Munich School of Management, Institute of Health Economics and Health Care Management, Ludwigstraße 28 1, 80539 Munich, Germany; 2 Institute of Health Economics and Health Care Management, HelmholtzZentrum München, Neuherberg, Germany; 3 AOK Bayern, Service Center of Health Care Management, Regensburg, Germany

**Keywords:** AMI, Health care expenditures, PDC, Secondary prevention, Guideline-based medication, DMP

## Abstract

**Background:**

Acute myocardial infarction (AMI), a major source of morbidity and mortality, is also associated with excess costs. Findings from previous studies were divergent regarding the effect on health care expenditure of adherence to guideline-recommended medication. However, gender-specific medication effectiveness, correlating the effectiveness of concomitant medication and variation in adherence over time, has not yet been considered.

**Methods:**

We aim to measure the effect of adherence on health care expenditures stratified by gender from a third-party payer’s perspective in a sample of statutory insured Disease Management Program participants over a follow-up period of 3-years. In 3627 AMI patients, the proportion of days covered (PDC) for four guideline-recommended medications was calculated. A generalized additive mixed model was used, taking into account inter-individual effects (mean PDC rate) and intra-individual effects (deviation from the mean PDC rate).

**Results:**

Regarding inter-individual effects, for both sexes only anti-platelet agents had a significant negative influence indicating that higher mean PDC rates lead to higher costs. With respect to intra-individual effects, for females higher deviations from the mean PDC rate for angiotensin-converting enzyme (ACE) inhibitors, anti-platelet agents, and statins were associated with higher costs. Furthermore, for males, an increasing positive deviation from the PDC mean increases costs for β-blockers and a negative deviation decreases costs. For anti-platelet agents, an increasing deviation from the PDC-mean slightly increases costs.

**Conclusion:**

Positive and negative deviation from the mean PDC rate, independent of how high the mean was, usually negatively affect health care expenditures. Therefore, continuity in intake of guideline-recommended medication is important to save costs.

**Supplementary Information:**

The online version contains supplementary material available at 10.1186/s12913-020-05946-4.

## Introduction

Although recent decades have seen improvements in mortality and survival rates [1], cardiovascular disease (CVD) remains one of the leading causes of mortality and morbidity in industrialized countries [2]. Acute myocardial infarction (AMI), a common manifestation of CVD in the elderly, carries increased risk of mortality, morbidity, and excess costs [3, 4]. In Germany, in 2016, 20,539 deaths in women and 28,130 deaths in men were caused by AMI, which reflected 49.2 and 69.3 deaths per 100,000 inhabitants in women and men respectively [5]. In the first year after AMI, cumulative total costs for AMI are about €13,061 per patient in Germany [6]. In the USA, the first-year costs are $17,532 for fatal and $15,540 for non-fatal AMI [7]. In the UK, between 0.4 and 1.0% of total health care expenditure was spent on AMI [8].

Worldwide, heart societies have released evidence-based guidelines for secondary prevention and management of AMI. Aside from lifestyle modifications, these guidelines encourage pharmacological therapy with anti-platelet agents, statins, β-blockers, and angiotensin-converting enzyme (ACE) inhibitors as long-term treatment [9, 10]. There is strong supporting evidence that re-infarction risk and patient mortality after AMI can be considerably reduced by using guideline-recommended medication [11–15]. However, studies have revealed discrepancies between recommended therapies and health care actually provided [16–23]. Notably, for all four drugs recommended after AMI in the guidelines, discontinuation of medical therapy is common, begins early after discharge, and increases substantially over time [18, 24, 25].

Non-adherence to medication is considered a major health policy issue, accounting for a considerable worsening of disease, poor prognosis, death, and increased health care costs [26, 27]. However, investigations on long-term medication adherence more than 1 year post AMI are scarce [25]. So far, three cross-sectional studies have been published measuring the influence of medication adherence on health care expenditures after AMI (focusing on renin–angiotensin system agents [28], statins [29], and statins and ACE inhibitors [30]). To date, no study exists with a longitudinal design that would allow capture of a variation in adherence rate over time or the influence of all guideline-recommended medications on health care expenditures simultaneously.

## Objective

The aim of this retrospective observational study is to measure the influence of adherence to guideline-recommended medication on health care expenditures in a real-world setting over a follow-up period of 3 years after AMI from the perspective of a third-party payer.

## Methods

### Data

The analysis is based on pseudonymized claims data routinely documented for participants in a Disease Management Program (DMP) for coronary artery disease (CAD). The data are provided by the AOK Bayern, a large regional health insurance fund in the south of Germany with a market share of more than 40%. The cost analysis was based on routine data on individual expenditures for filed claims, including the categories of hospital, outpatient care, medication, rehabilitation (if covered by AOK Bayern), and costs for remedial and aid products. According to the ethics committee of the State Chamber of Physicians of Bavaria, no ethical approval was required.

### Study population

Individuals were included in the study if they had at least one hospitalization with a main discharge diagnosis of AMI (ICD-10 I21) between January 1, 2009 and December 31, 2011. AMI patients before 2009 were excluded because hierarchical morbidity group (HMG) compensation, which was used as a control variable for morbidity, was not available before 2009. AMI patients after 2011 were excluded as the 3-year follow-up period would not be covered by the data available. Further inclusion criteria were that patients had to be enrolled in the DMP CAD before the inception hospitalization and continuously insured at least 1 year before and 3 years after hospitalization, unless they died. Patients were excluded if documentation for the DMP CAD was missing in the 180 days prior to the AMI, if they died within 30 days after the first hospitalization, or if they had missing values of covariates.

### Medication

Adherence to guideline-based secondary prevention for AMI [31] was assessed through the anatomical therapeutic chemical (ATC) classification system for anti-platelet agents (B01A), statins (C10), β-blockers (C07), and ACE inhibitors (C09A and C09B).

### Adherence

Proportion of days covered (PDC) was calculated for the year before AMI and for each year in the 3-year follow-up period. To this end, we calculated the total number of days supplied in each period based on the number of prescriptions multiplied by the defined daily dose (DDD) per prescription. If the DDDs of a prescription extended into a new period, they were considered as medication stock in this period. DDDs were supplied by the scientific institute of the AOK (WIdO) based on a German adaptation of the WHO database. If there were discrepancies between the WIdO DDDs and the DDD recommendations of the national guidelines [32] (see also Online Additional file 1: Table 1), then the dosage from the national guidelines was used. DDD adjustments were made for ACE inhibitors (Enalapril: 20 mg instead of 10 mg (C09AA02), Perindopril: 8 mg instead of 4 mg (C09AA04), Ramipril: 10 mg instead of 2.5 mg (C09AA05), Quinapril: 20 mg instead of 15 mg (C09AA06), and Trandolapril: 4 mg instead of 2 mg (C09AA10)), and statins (Simvastatin: 40 mg instead of 30 mg (C10AA01), Lovastatin: 40 mg instead of 45 mg (C10AA02), Pravastatin: 40 mg instead of 30 mg (C10AA03), and Atorvastatin: 10 mg instead of 20 mg (C10AA05)). For β-blockers, national guidelines [32] recommend intake only for 1 up to 2 years after the AMI. Therefore, adherence was determined by the PDC in the first year after AMI.

### Outcome measures

Primary outcome measures were the average overall health care expenditures per year, including ambulatory, medication, hospitalization, rehabilitation, and remedial and aid costs. Further analyses were conducted for every single cost category. All costs were inflated to 2014 euros, using the inflation rate reported for Germany by the OECD [33].

### Statistical analysis

We stratified the AMI patients for sex in our analysis as there are gender-specific similarities in the effectiveness of anti-platelet agents [34, 35] and statins [36, 37], but differences in β-blockers [38–40] and ACE inhibitors [41–43], which should also be reflected in costs. Characteristics of patients stratified by sex in the 3 years after AMI were compared (Table 1) using analysis of variance (ANOVA) for continuous variables and Chi^2^ tests for categorical variables. We examined the association between health care expenditures and PDC rates (PDC mean and PDC standard deviation) for anti-platelet agents, statins, β-blockers, and ACE inhibitors. Considering points in time or periods (e.g., year 1 of observation after AMI) as nested in individuals, the analysis divided the original independent variable (PDC rates) into the mean over time (between-subject change or inter-personal effect) and deviation from the mean over time (within-subject change or intra-personal effect). Specifically, the analysis distinguished between the cross-sectional inter-personal (PDC mean estimates) and the longitudinal intra-personal (PDC standard deviation) associations of PDC rates (included as continuous variables in percentage difference or change) on health care expenditures [44]. We estimated these effects with a generalized additive mixed model (GAMM) with a smoothing function of PDC rates (PDC mean and standard deviation). A GAMM is a generalized linear mixed model in which the linear predictor depends linearly on unknown smooth functions of the covariates of interest. For the smooth function, penalized regression spline type smoothers of moderate rank are used. For estimation purposes, the generalized component of each smooth is treated as a random effect term, while the unpenalized component is treated as fixed [45, 46]. Linear mixed models are an extension of simple linear models to allow for both fixed and random effects, and are regularly used if there is no independence in the data, which may arise from a hierarchical structure or repeated measurement. Patient-level observations over several years are not independent from each other. For this reason, we added random intercepts for each patient. In this way, we estimate patient-specific intercepts, as each patient will have their own unique effect added to the overall intercept. In general, the interpretation of results from a GAMM is similar to an ordinary linear mixed model. The main difference is that, for the smooth terms, there is no single coefficient you can make inference from (i.e., negative, positive, effect size, etc.). Hence, one needs to rely on interpreting the partial effects of the smooth terms visually.

**Table 1:** Descriptive statistics by sex and year

		Year 1 after AMI			Year 2 after AMI			Year 3 after AMI		
		Male	Female		Male	Female		Male	Female	
**N**^**b**^		2440 (67.40%)	1180 (32.60%)	***	2066 (68.73%)	940 (31.27%)	***	1845 (69.33%)	816 (30.67%)	***
**Age**^**a**^		71.67 (10.23)	77.60 (9.05)	***	72.03 (10.13)	77.60 (9.02)	***	72.35 (10.10)	77.92 (9.05)	***
**Age groups**^**b**^	**< 55**	184 (7.54%)	28 (2.37%)	***	150 (7.26%)	24 (2.55%)	***	125 (6.78%)	22 (2.70%)	***
	**≥ 55 < 65**	397 (16.27%)	79 (6.69%)		333 (16.12%)	62 (6.60%)		295 (15.99%)	52 (6.37%)	
	**≥ 65 < 75**	851 (34.88%)	291 (24.66%)		700 (33.88%)	238 (25.32%)		609 (33.01%)	193 (23.65%)	
	**≥ 75**	1008 (41.31%)	782 (66.27%)		883 (42.74%)	616 (65.53%)		816 (44.23%)	549 (67.28%)	
**BMI groups**^**b**^	**< 18.5**	5 (0.20%)	20 (1.69%)	***	5 (0.24%)	12 (1.28%)	***	3 (0.16%)	8 (0.98%)	***
	**≥ 18.5 < 25**	452 (18.52%)	309 (26.19%)		382 (18.49%)	235 (25.00%)		333 (18.05%)	204 (25.00%)	
	**≥ 25 < 30**	1203 (49.30%)	471 (39.92%)		1002 (48.50%)	362 (38.51%)		869 (47.10%)	300 (36.76%)	
	**≥ 30**	780 (31.97%)	380 (32.20%)		677 (32.77%)	331 (35.21%)		640 (34.69%)	304 (37.25%)	
**BIMD 2010 (Q1 least deprived, Q5 most deprived)**^**b**^	**Quartile 1**	471 (19.30%)	211 (17.88%)		398 (19.26%)	164 (17.45%)		356 (19.30%)	144 (17.65%)	
	**Quartile 2**	552 (22.62%)	261 (22.12%)		483 (23.38%)	215 (22.87%)		445 (24.12%)	191 (23.41%)	
	**Quartile 3**	429 (17.58%)	214 (18.14%)		346 (16.75%)	168 (17.87%)		301 (17.29%)	141 (17.28%)	
	**Quartile 4**	433 (17.75%)	229 (19.41%)		363 (17.57%)	187 (19.89%)		319 (17.29%)	159 (19.49%)	
	**Quartile 5**	555 (22.75%)	265 (22.46%)		476 (23.04%)	206 (21.91%)		424 (22.98%)	181 (22.18%)	
**Smoking status**^**b**^	**Smoker**	344 (14.10%)	88 (7.46%)	***	292 (14.13%)	66 (7.02%)	***	254 (13.77%)	60 (7.35%)	***
**NYHA**^**b**^	**0**	1216 (49.84%)	509 (43.14%)	***	991 (47.97%)	401 (42.66%)	*	852 (46.18%)	331 (40.56%)	*
	**1**	71 (2.91%)	35 (2.97%)		69 (3.34%)	31 (3.30%)		68 (3.69%)	30 (3.68%)	
	**2**	281 (11.52%)	131 (11.10%)		267 (12.92%)	132 (14.04%)		262 (14.20%)	123 (15.07%)	
	**3**	446 (18.28%)	226 (19.15%)		397 (19.22%)	183 (19.47%)		381 (20.65%)	172 (21.08%)	
	**4**	426 (17.46%)	279 (23.64%)		342 (16.55%)	193 (20.53%)		282 (15.28%)	160 (19.61%)	
**DMP COPD**^**b**^		214 (8.77%)	68 (5.76%)	**	186 (9.00%)	53 (5.64%)	**	157 (8.51%)	43 (5.27%)	**
**DMP asthma**^**b**^		54 (2.21%)	36 (3.05%)		47 (2.27%)	32 (3.40%)		38 (2.06%)	31 (3.80%)	**
**DMP type 1 diabetes**^**b**^		7 (0.29%)	5 (0.42%)		5 (0.24%)	5 (0.53%)		4 (0.22%)	4 (0.49%)	
**DMP type 2 diabetes**^**b**^		1048 (42.95%)	564 (47.80%)	**	907 (43.90%)	450 (47.87%)	*	818 (44.34%)	392 (48.04%)	
**Death in observation period**^**b**^		252 (10.33%)	151 (12.80%)	*	145 (7.02%)	79 (8.40%)		113 (6.12%)	62 (7.60%)	
**HMG assignment per month**^**a**^		€479.24 (€652.99)	€505.28 (€552.82]		€757.68 (€768,39)	€786.08 (€656.48)		€580.85 (€717.10)	€555.54 (€539.76)	
**Days survived in observation period**^**a**^		347.10 (60.87)	342.19 (69.79)	*	354.07 (48.19)	351.78 (52.95)		355.31 (45.53)	353.57 (48.04)	
**Angina pectoris**^**b**^		1122 (45.98%)	510 (43.22%)		441 (21.35%)	191 (20.32%)		881 (47.75%)	385 (47.18%)	
**Peripheral vascular disease**^**b**^		2332 (95.57%)	1057 (89.58%)	***	1407 (68.10%)	588 (62.55%)	**	1784 (96.69%)	754 (92.40%)	***
**Dyslipidemia**^**b**^		2071 (84.88%)	937 (79.41%)	***	1560 (75.51%)	650 (69.15%)	***	1620 (87.80%)	680 (83.33%)	**
**Congestive heart failure**^**b**^		1385 (56.76%)	758 (64.24%)	***	868 (42.01%)	453 (48.19%)	**	938 (50.84%)	471 (57.72%)	**
**Hypertension**^**b**^		2341 (95.94%)	1137 (96.36%)		1819 (88.04%)	840 (89.36%)		1780 (96.48%)	790 (96.81%)	
**Dialysis**^**b**^		81 (3.32%)	27 (2.29%)		63 (3.05%)	17 (1.81%)		55 (2.98%)	12 (1.47%)	*
**PDC-rate ACE inhibitors**^**a**^		72.41% (40.13)	66.96 (42.97)	***	69.16 (42.40)	61.94 (45.57)	***	67.42 (43.88)	60.23 (46.37)	***
**PDC-rate β-blockers**^**a**^		47.76 (32.30)	48.03 (33.86)		43.40 (33.14)	43.55 (34.22)		42.75 (33.58)	43.64 (35.37)	
**PDC-rate statins**^**a**^		83.69 (32.19)	75.10 (39.70)	***	82.68 (33.53)	73.54 (40.79)	***	80.75 (35.56)	72.40 (41.81)	***
**PDC-rate anti-platelet agents**^**a**^		46.42 (41.60)	44.65 (41.16)		32.83 (39.99)	31.78 (38.99)		27.02 (37.97)	25.41 (35.51)	
**Health care expenditures**^**a**^		€18,467.68 (€18,775.49)	€16,846.24 (€15,868.08)	**	€5723.61 (€10,33.65)	€5549.28 (€10,266.74)		€5582.88 (€10,598.59)	€5430.74 (€9481.66)	

^a^mean (SD)

^b^absolute numbers (percentages)

^a^*p*-value based on ANOVA

^b^p-value based on Chi^2^-test

Significant differences between the group 0 drugs and the other groups:* *p* < 0.05 ** *p* < 0.01 *** *p* < 0.001

*Abbreviations*: *BIMD 2010* (Bavarian Index of Multiple Deprivation, year 2010), *BMI* (Body Mass Index), *DMP* (Disease Management Program), *HMG* (Hierarchical Morbidity Group), *NYHA* (New York Hear Association), *PDC* (Proportion of days covered))

The GAMM was adjusted for age (< 55, 55 < 65, 65 < 75, and ≥ 75 years), Body-Mass Index (BMI) (underweight, normal weight, overweight, and obesity), smoking status, New York Heart Association classification (NYHA) (no NYHA, NYHA 1 to NYHA 4), enrollment in the DMPs for chronic obstructive pulmonary disease (COPD), asthma, type 1 diabetes, type 2 diabetes, death in observation period, HMG assignment per month, year of observation following the AMI (year 1, year 2, and year 3), days survived in the year of observation, angina pectoris (ICD-10: I20), peripheral vascular disease (ICD-10: I25), dyslipidemia (ICD-10: E78), congestive heart failure (ICD-10: I50), hypertension (ICD-10: I11–I15), and dialysis (patients were identified as dialysis patients if they had incurred dialysis costs according to data from the statutory health insurance fund). Additionally, owing to the absence of data on individual socio-economic status, the Bavarian Index of Multiple Deprivation (BIMD) 2010, subdivided into quintiles reaching from least (Q1) to most (Q5) deprived districts in Bavaria, was used as a proxy [47, 48]. The index was developed as a small-area, multidimensional deprivation index based on an established British method [49] and combines official sociodemographic, socioeconomic, and environmental data in seven domains of deprivation. Furthermore, to estimate the influence of each single cost category on total health care expenditures, we also conducted the same analysis used for total health care expenditure separately for each of the cost categories that were included in the total health care expenditures (i.e., ambulatory, medication, hospitalization, rehabilitation, and remedial and aid costs).

### Sensitivity analysis

To analyze the robustness of the results two further analyses were conducted. First, patients spending more than 50% of the follow-up time in hospital were excluded and, second, only patients surviving the 3-year follow-up period were considered.

The GAMM was estimated using the statistical software R (version 3.5.1) and applying the gamm4 package [45].

## Results

### Total health care expenditure

**Fig. 1:**
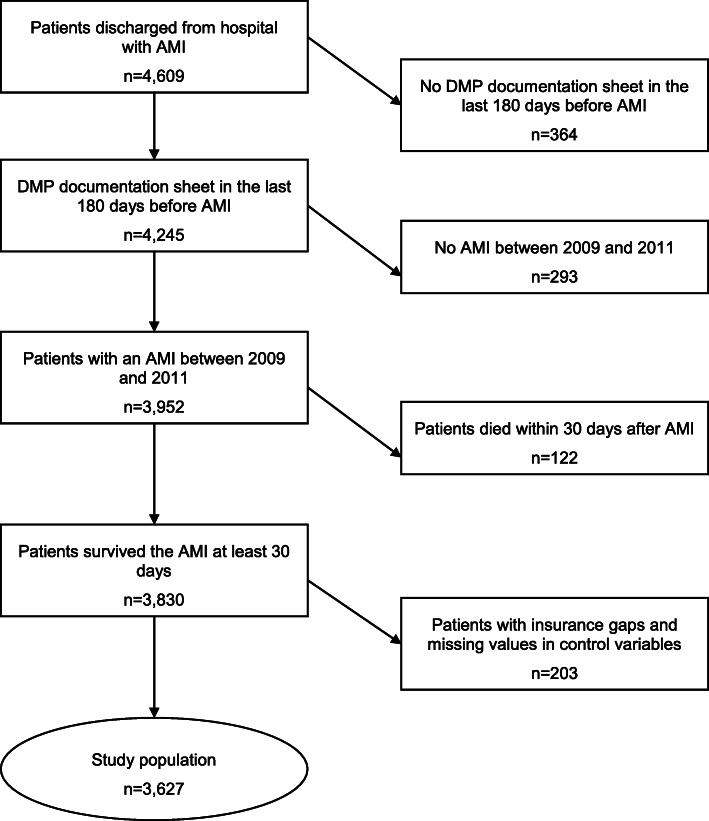
Patient selection

The data set consisted of 4609 DMP CAD patients discharged from hospital with a diagnosis of AMI, of which 4245 had a complete DMP documentation sheet in the last 180 days before AMI. Out of this group, 3952 patients had an AMI in the period between January 1, 2009 and December 31, 2011. Of these, 122 people died within 30 days, and another 203 people were excluded because of insurance gaps or missing data. Hence, the study population comprised 3627 patients (Fig. 1).

Baseline characteristics are presented in Table 1. In total, observations of 3620 (1180 female and 2440 male), 3006 (940 female and 2066 male), and 2661 (816 female and 1845 male) subjects were considered in years 1, 2, and 3 respectively. On average, males were more than 5 years younger (*p* < 0.001), had a higher BMI (p < 0.001), and the percentage of active smokers was approximately twice as high (p < 0.001). Regarding heart-related comorbidities, males were less often in a higher NYHA state (*p* < 0.05) and suffered less from congestive heart failure (*p* < 0.01), but they had higher rates of dyslipidemia (p < 0.01) and peripheral vascular disease (p < 0.01). Besides this, the percentage of males enrolled in the DMP type 2 diabetes was lower in the first (p < 0.01) and second year (*p* < 0.05), and the percentage of males who died in the first year after AMI was lower (p < 0.05), leading to a higher number of days survived in the first year (p < 0.05). PDC rates in males were higher on ACE inhibitors (*p* < 0.001) and statins (p < 0.001) but similar on β-blockers and anti-platelet agents.

**Fig. 2:**
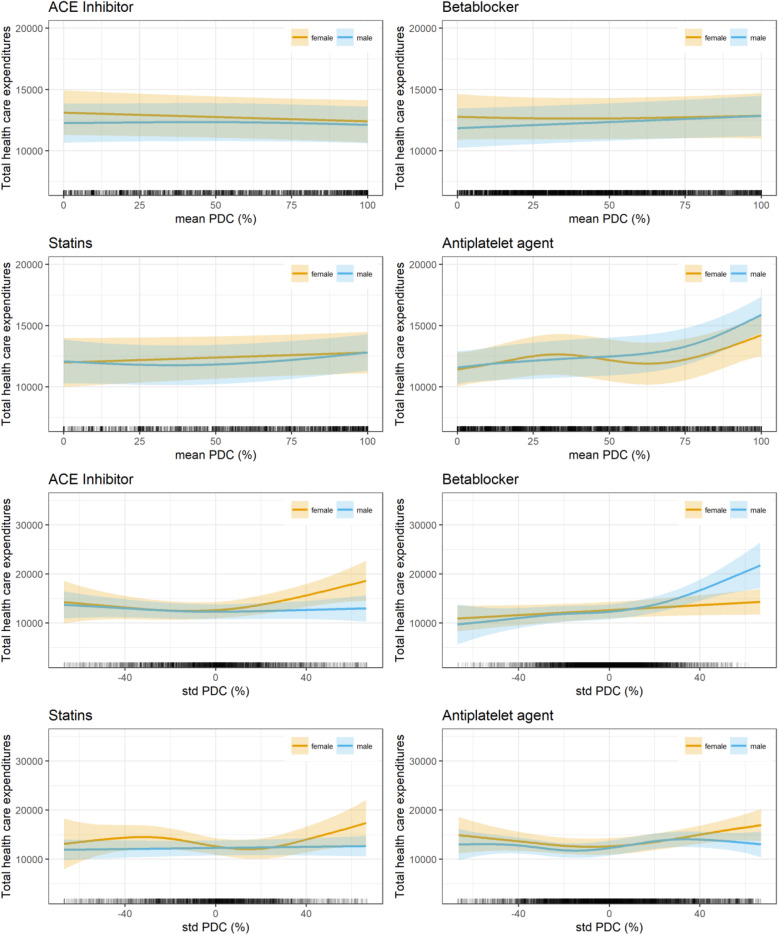
Base case – Influence on total health care expenditures

The results of the GAMM on health care expenditures are presented in Table 2 and Fig. 2. In the model (Table 2), the highest age group and patients living in the least deprived districts are associated with lowest costs (*p* < 0.05). Furthermore, enrollment in the DMP COPD (p < 0.05), a more severe NYHA state (*p* < 0.01), the occurrence of comorbidities angina pectoris (*p* < 0.001), peripheral vascular disease (p < 0.001), congestive heart failure (*p* < 0.001), hypertension (p < 0.01), and being a dialysis patient (p < 0.001) were associated with higher costs. Similarly, death (p < 0.001), the number of days insured (p < 0.001) in the observation period, and higher HMG assignments per month (p < 0.01) were associated with higher costs. Contrarily, costs decreased in the consecutive years after AMI (*p* < 0.001).

**Table 2:** Base Case - Influence of PDC rates on health care expenditures

***N*** = 9287		Estimate	Std. Error	t value	Pr(>|t|)
**(Intercept)**		− 1089.26	1916.40	−0.57	0.5698
**Age**	**55 < 65**	611.05	687.99	0.89	0.3745
	**65 < 75**	323.90	642.00	0.50	0.6139
	**≥ 75**	− 1481.82	647.83	−2.29	0.0222*
**Gender**	**Female**	−147.47	309.84	−0.48	0.6341
**BMI**	**underweight**	1076.18	1814.31	0.59	0.5531
	**overweight**	−287.73	365.01	−0.79	0.4306
	**obese**	−407.75	395.78	−1.03	0.3029
**BIMD 2010 (Q1 least deprived, Q5 most deprived)**	**Q2**	− 600.25	427.80	−1.40	0.1606
	**Q3**	− 935.95	459.42	− 2.04	0.0417*
	**Q4**	− 535.59	454.13	−1.18	0.2383
	**Q5**	− 812.27	430.76	−1.89	0.0594
**Smoker**	**yes**	157.39	447.42	0.35	0.7250
**NYHA**	**1**	2552.83	810.15	3.15	0.0016**
	**2**	1548.90	482.24	3.21	0.0013**
	**3**	2887.35	434.52	6.64	0.0000***
	**4**	4533.88	457.35	9.91	0.0000***
**DMP COPD**	**yes**	1206.22	517.34	2.33	0.0197*
**DMP asthma**	**yes**	− 829.61	872.64	−0.95	0.3418
**DMP diabetes type 1**	**yes**	1946.15	2436.29	0.80	0.4244
**DMP diabetes type 2**	**yes**	519.76	288.19	1.80	0.0713
**deceased**	**yes**	11,012.39	925.20	11.90	0.0000***
**HMG assignments per month**		1.09	0.24	4.45	0.0000***
**Year after AMI**		− 5914.40	168.43	−35.11	0.0000***
**days insured**		34.48	4.72	7.31	0.0000***
**Angina pectoris**		2115.66	280.69	7.54	0.0000***
**Peripheral vascular disease**		4415.08	396.30	11.14	0.0000***
**Dyslipidemia**		480.99	365.24	1.32	0.1879
**Congestive heart failure**		2074.91	352.90	5.88	0.0000***
**Hypertension**		1939.37	574.43	3.38	0.0007***
**Dialysis**		23,554.62	991.15	23.77	0.0000***
		**edf**	**Ref.df**	**F**	**p-value**
**s (PDC mean ACE inhibitors) male**		1.26	1.26	0.09	0.7234
**s (PDC mean ACE inhibitors) female**		1.00	1.00	1.45	0.2292
**s (PDC mean β-blockers) male**		1.00	1.00	3.01	0.0827
**s (PDC mean β-blockers) female**		1.29	1.29	0.12	0.8398
**s (PDC mean statins) male**		1.78	1.78	1.98	0.0851
**s (PDC mean statins) female**		1.00	1.00	1.43	0.2310
**s (PDC mean anti-platelet agents) male**		2.99	2.99	21.63	0.0000***
**s (PDC mean anti-platelet agents) female**		3.08	3.08	4.34	0.0055**
**s (PDC standard deviation ACE inhibitors) male**		1.90	1.90	0.97	0.4112
**s (PDC standard deviation ACE inhibitors) female**		2.51	2.51	5.11	0.0038**
**s (PDC standard deviation β-blockers) male**		3.25	3.25	12.01	0.0000***
**s (PDC standard deviation β-blockers) female**		1.00	1.00	2.77	0.0960
**s (PDC standard deviation statins) male**		1.00	1.00	0.26	0.6070
**s (PDC standard deviation statins) female**		3.35	3.35	2.97	0.0368*
**s (PDC standard deviation anti-platelet agents) male**		3.99	3.99	4.23	0.0023**
**s (PDC standard deviation anti-platelet agents) female**		2.69	2.69	4.04	0.0116*

R-sq. (adj.) = 0.324

*Abbreviations*: *AMI* (Acute Myocardial infarction), *BIMD 2010* (Bavarian Index of Multiple Deprivation, year 2010), *BMI* (Body Mass Index), *DMP* (Disease Management Program), *HMG* (Hierarchical Morbidity Group), *NYHA* (New York Hear Association), *PDC* (Proportion of days covered)

Significane Levels:* *p* < 0.05 ** *p* <0.01 *** *p* < 0.001

In Fig. 2, the height of the mean PDC rates (inter-personal effect) in the complete observation period after AMI for ACE inhibitors, β-blockers, and statins did not seem to influence health care expenditures in females or males. Only for anti-platelet agents was a negative effect of a higher PDC rate on health care expenditures found for females (*p* < 0.05) and males (*p* < 0.001). The intra-personal effect (positive or negative deviation from a person’s mean PDC-rate) seemed to have a greater effect on health care expenditures for ACE inhibitors in females (*p* < 0.01), for β-blockers in males (p < 0.001), for statins in females (*p* < 0.05), and for anti-platelet agents in both females (p < 0.01) and males (p < 0.001). For ACE inhibitors and β-blockers in females, an increasing negative or positive deviation from the mean PDC rate was associated with higher costs. For statins (p < 0.05), first an increase and then a decrease in health care expenditures for an increasing negative deviation and an increase in health care expenditures for an increasing positive deviation from the mean in females were observed. In males, an increasing negative deviation from the PDC mean in β-blockers reduced the costs and an increasing positive deviation increased costs. For anti-platelet agents in males, an increasing negative deviation from the mean was associated with higher costs, and an increasing positive deviation first increased and then decreased costs.

### Individual categories of health care expenditures

The analyses on individual cost categories (ambulatory, medication, hospitalization, rehabilitation, and remedial and aid costs) were conducted for the base case. Similar to the analysis of total health care expenditures for all individual cost categories, the intra-individual effect had more influence on costs than the inter-individual effect. We consider the different cost categories in turn.

With regard to ambulatory costs (see Online Table 1 and Online Figure 1), the inter-individual effect was relevant for ACE inhibitors and anti-platelet agents for females, and the intra-individual effect for ACE inhibitors in females and for β-blockers in both sexes. More specifically, higher mean PDC rates for males in ACE inhibitors and β-blockers were associated with increased costs, but this was the case for anti-platelet agents for females. With the exception of statins in females, higher positive deviations from the mean PDC rate were associated with decreased ambulatory costs.

Regarding medication costs (see Online Table 2 and Online Figure 2), inter-individual effects were important for ACE inhibitors, statins, and anti-platelet agents in females and for β-blockers and anti-platelet agents in males. The intra-individual effect was of relevance for all medications in females and for β-blockers and anti-platelet agents in males. Increasing PDC means were associated with increasing medication costs for β-blockers, statins, and anti-platelet agents in males and anti-platelet agents in females. A higher positive deviation from the mean PDC rate was associated with increased costs for β-blockers and anti-platelet agents in both males and females.

With respect to hospitalization costs (see Online Table 3 and Online Figure 3), the inter-individual effect was only relevant for anti-platelet agents in males, and the intra-individual effect was important for ACE inhibitors and anti-platelet agents in females and for β-blockers and anti-platelet agents in males. More precisely, a higher mean PDC rate was associated with higher hospitalization costs for β-blockers, statins, and anti-platelet agents in both males and females. A higher positive deviation from the mean was associated with higher hospitalization costs for β-blockers in males and for all medications in females.

For rehabilitation costs (see Online Table 4 and Online Figure 4), the inter-individual effect was only remarkable in males for statins. The intra-individual effect was relevant for ACE inhibitors in females and for anti-platelet agents in both sexes. Specifically, a higher mean PDC rate was associated with higher rehabilitation expenditures for statins and anti-platelet agents in both sexes, and a higher positive deviation from the mean PDC rate was associated with higher costs for ACE inhibitors, β-blockers, and anti-platelet agents in males and ACE inhibitors, statins, and anti-platelet agents in females.

Finally, for remedial and aid costs (see Online Table 5 and Online Figure 5), the inter-individual effect was only important for statins and anti-platelet agents in males. The intra-individual effect was of relevance for ACE inhibitors, β-blockers, and anti-platelet agents in females and for β-blockers in males. Although a higher mean PDC rate was associated with higher costs only for anti-platelet agents in males and females, a positive deviation from the mean PDC rate was associated with higher expenditures for β-blockers and anti-platelet agents in both sexes and also for ACE inhibitors and statins in females.

### Sensitivity analysis

**Fig. 3:**
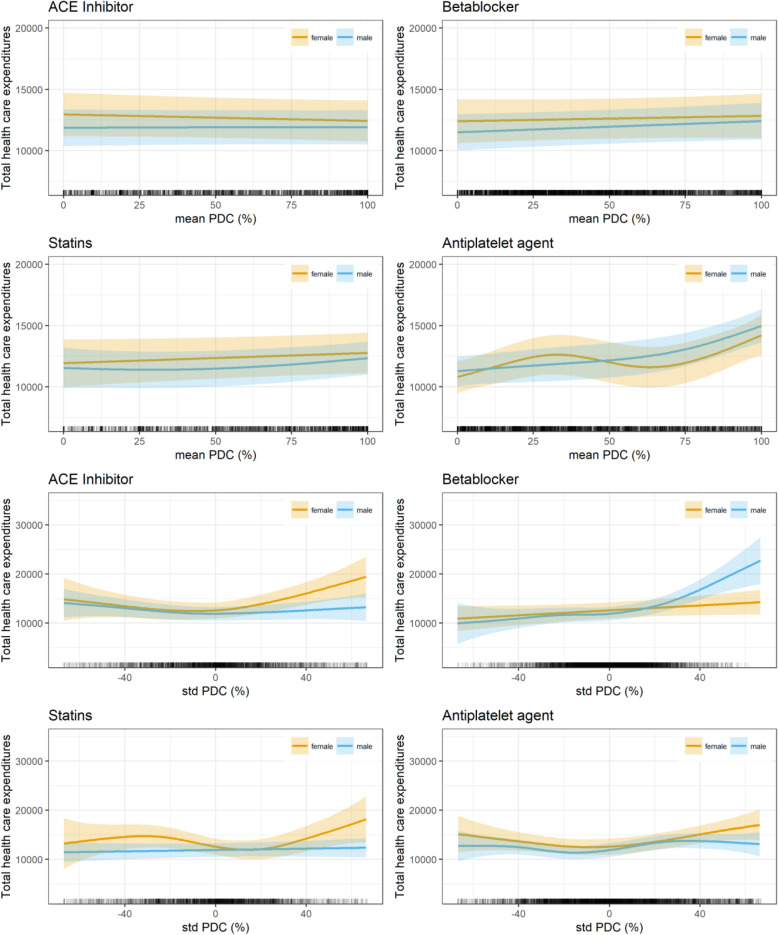
Sensitivity analysis 1 – Influence on total health care expenditures

In the first sensitivity analysis (Table 3 and Fig. 3), all patients who spent more than 50% of the observed days in hospital were excluded. This led to 9147 observations in the 3 years after AMI. Despite very few minor changes with respect to some covariates, the results were quite similar to the base case analysis. Although the significance levels changed slightly, no differences from the base case were found for the association between PDC mean and deviation from the PDC mean and health care expenditures. Accordingly, the shapes of the curves remained almost exactly the same as in the base case analysis.

**Table 3:** Sensitivity analysis 1 – Influence of PDC rates on health care expenditures

***N*** = 9147		Estimate	Std. Error	t value	Pr(>|t|)
**(Intercept)**		− 4388.13	1956.20	−2.24	0.0249*
**Age**	**55 < 65**	366.80	667.03	0.55	0.5824
	**65 < 75**	23.96	622.49	0.04	0.9693
	**≥ 75**	− 1479.16	628.26	−2.35	0.0186*
**Gender**	**female**	− 182.06	298.09	−0.61	0.5414
**BMI**	**underweight**	760.97	1784.07	0.43	0.6697
	**overweight**	−105.89	352.11	−0.30	0.7636
	**obese**	− 177.73	380.95	−0.47	0.6408
**BIMD 2010 (Q1 least deprived, Q5 most deprived)**	**Q2**	− 342.96	410.99	− 0.83	0.4040
	**Q3**	−745.28	441.27	−1.69	0.0913
	**Q4**	− 299.33	436.92	−0.69	0.4933
	**Q5**	− 781.20	414.84	−1.88	0.0597
**Smoker**	**yes**	−49.90	431.94	−0.12	0.9080
**NYHA**	**1**	2580.50	776.13	3.32	0.0009***
	**2**	1378.37	463.25	2.98	0.0029**
	**3**	2809.18	417.38	6.73	0.0000***
	**4**	4422.92	440.04	10.05	0.0000***
**DMP COPD**	**yes**	1042.85	498.59	2.09	0.0365*
**DMP asthma**	**yes**	− 759.08	841.90	− 0.90	0.3673
**DMP diabetes type 1**	**yes**	2033.06	2326.11	0.87	0.3821
**DMP diabetes type 2**	**yes**	505.34	276.79	1.83	0.0679
**deceased**	**yes**	10,472.27	911.95	11.48	0.0000***
**HMG assignments per month**		1.23	0.24	5.18	0.0000***
**Year after AMI**		− 5722.92	162.81	−35.15	0.0000***
**days insured**		42.73	4.86	8.79	0.0000***
**Angina pectoris**		2027.02	270.66	7.49	0.0000***
**Peripheral vascular disease**		4179.64	382.42	10.93	0.0000***
**Dyslipidemia**		498.35	352.61	1.41	0.1576
**Congestive heart failure**		2090.90	339.30	6.16	0.0000***
**Hypertension**		1867.35	556.62	3.35	0.0008***
**Dialysis**		23,977.62	964.93	24.85	0.0000***
		**edf**	**Ref.df**	**F**	**p-value**
**s (PDC mean ACE inhibitors) male**		1.04	1.04	0.01	0.9423
**s (PDC mean ACE inhibitors) female**		1.00	1.00	0.89	0.3465
**s (PDC mean β-blockers) male**		1.00	1.00	2.70	0.1003
**s (PDC mean β-blockers) female**		1.00	1.00	0.32	0.5704
**s (PDC mean statins) male**		1.65	1.65	1.87	0.0949
**s (PDC mean statins) female**		1.00	1.00	1.61	0.2044
**s (PDC mean anti-platelet agents) male**		2.49	2.49	18.84	0.0000***
**s (PDC mean anti-platelet agents) female**		3.38	3.38	6.26	0.0003***
**s (PDC standard deviation ACE inhibitors) male**		2.35	2.35	1.73	0.1356
**s (PDC standard deviation ACE inhibitors) female**		2.72	2.72	6.54	0.0005***
**s (PDC standard deviation β-blockers) male**		3.63	3.63	12.74	0.0000***
**s (PDC standard deviation β-blockers) female**		1.00	1.00	2.89	0.0893
**s (PDC standard deviation statins) male**		1.00	1.00	0.43	0.5129
**s (PDC standard deviation statins) female**		3.58	3.58	3.73	0.0107*
**s (PDC standard deviation anti-platelet agents) male**		4.05	4.05	4.77	0.0007***
**s (PDC standard deviation anti-platelet agents) female**		2.79	2.79	4.54	0.0059**

R-sq. (adj.) = 0.333

*Abbreviations*: *AMI* (Acute Myocardial infarction), *BIMD 2010* (Bavarian Index of Multiple Deprivation, year 2010), *BMI* (Body Mass Index), *DMP* (Disease Management Program), *HMG* (Hierarchical Morbidity Group), *NYHA* (New York Hear Association), *PDC* (Proportion of days covered)

Significane Levels:* *p* < 0.05 ** *p* <0.01 *** *p* < 0.001

**Fig. 4:**
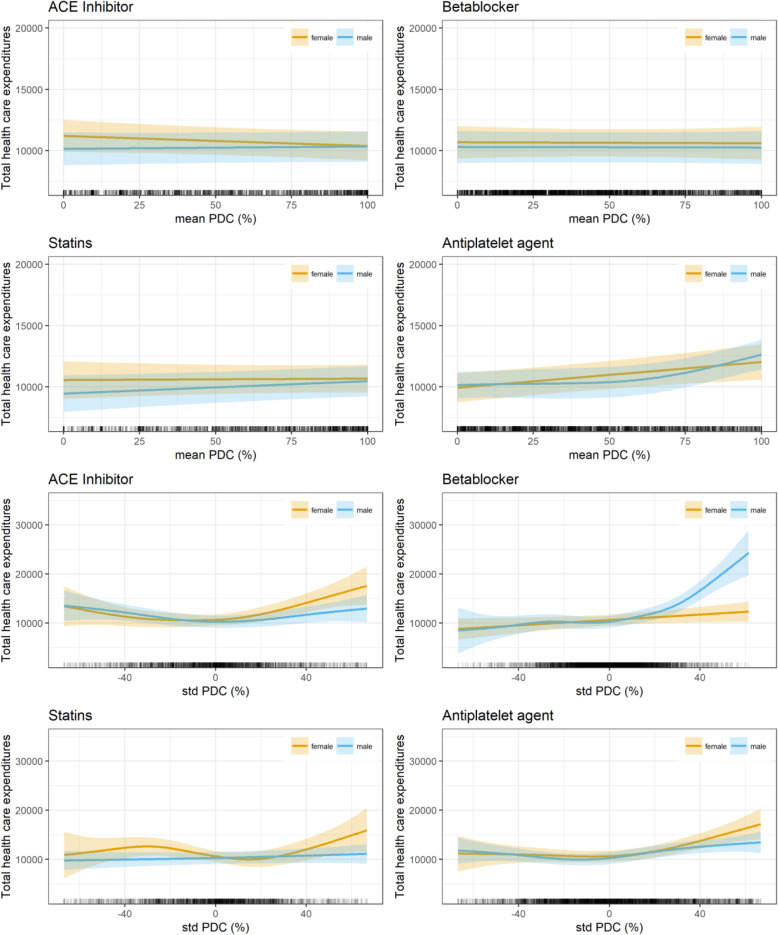
Sensitivity analysis 2 – Influence on total health care expenditures

In the second sensitivity analysis (Table 4 and Fig. 4), all patients who died in the observation period were excluded, leading to 7532 observations in the 3 years after AMI. Therefore, the variables “deceased” and “number of days in the observation period” were removed from the model. In contrast to the first sensitivity analysis, some more differences from the base case analysis could be detected. Regarding the covariates, health care costs in the fifth, but not in the third, quintile of the BIMD 2010 (*p* < 0.05) are significantly lower than in the first quintile, whereas dyslipidemia (p < 0.05) increased health care expenditures. Contrarily, the effect of enrollment in the DMP type 2 diabetes was not significant any more. However, the shape of the curves for mean PDC rate and deviation from the mean PDC rate were quite similar to the base case. PDC means for statins in males (*p* < 0.001) and females (p < 0.05) and a deviation from mean PDC rates in ACE inhibitors (*p* < 0.01), statins (p < 0.05), and anti-platelet agents (p < 0.001) in females and β-blockers (p < 0.001) and anti-platelet agents (p < 0.001) in males remained significant. Additionally to the base case, the intra-individual effect for ACE inhibitors (p < 0.01) in males becomes significant and indicates that a positively or negatively increasing deviation from the PDC mean also increases costs health care expenditures.

**Table 4:** Sensitivity analysis 2 - Influence of PDC rates on health care expenditures

***N*** = 7532		Estimate	Std. Error	t value	Pr(>|t|)
**(Intercept)**		10,032.21	840.93	11.93	0.0000***
**Age**	**55 < 65**	−105.27	573.67	−0.18	0.8544
	**65 < 75**	−44.58	537.60	−0.08	0.9339
	**≥ 75**	− 1067.64	546.18	−1.95	0.0507
**Gender**	**female**	−58.70	274.20	−0.21	0.8305
**BMI**	**underweight**	1949.13	1756.07	1.11	0.2671
	**overweight**	− 228.12	331.73	−0.69	0.4917
	**obese**	− 140.00	352.75	−0.40	0.6915
**BIMD 2010 (Q1 least deprived, Q5 most deprived)**	**Q2**	−505.20	371.50	−1.36	0.1739
	**Q3**	− 738.01	404.06	−1.83	0.0678
	**Q4**	− 545.68	398.88	−1.37	0.1713
	**Q5**	−768.79	376.58	−2.04	0.0412*
**Smoker**	**yes**	183.30	387.00	0.47	0.6358
**NYHA**	**1**	1852.93	707.26	2.62	0.0088**
	**2**	1539.45	414.21	3.72	0.0002***
	**3**	2743.89	381.06	7.20	0.0000***
	**4**	4034.88	422.76	9.54	0.0000***
**DMP COPD**	**yes**	310.39	477.84	0.65	0.5160
**DMP asthma**	**yes**	− 681.41	731.89	−0.93	0.3519
**DMP diabetes type 1**	**yes**	2067.03	1966.07	1.05	0.2931
**DMP diabetes type 2**	**yes**	424.88	253.61	1.68	0.0939
**HMG assignments per month**		1.41	0.24	5.77	0.0000***
**Year after AMI**		− 5133.93	155.94	−32.92	0.0000***
**Angina pectoris**		1766.07	249.36	7.08	0.0000***
**Peripheral vascular disease**		3727.85	358.26	10.41	0.0000***
**Dyslipidemia**		737.41	334.28	2.21	0.0274*
**Congestive heart failure**		1761.57	308.12	5.72	0.0000***
**Hypertension**		1925.66	520.03	3.70	0.0002***
**Dialysis**		25,186.07	1035.41	24.32	0.0000****
		**edf**	**Ref.df**	**F**	**p-value**
**s (PDC mean ACE inhibitors) male**		1.00	1.00	0.26	0.6135
**s (PDC mean ACE inhibitors) female**		1.00	1.00	2.44	0.1179
**s (PDC mean β-blockers) male**		1.00	1.00	0.01	0.9286
**s (PDC mean β-blockers) female**		1.00	1.00	0.01	0.9118
**s (PDC mean statins) male**		1.00	1.00	3.84	0.0500
**s (PDC mean statins) female**		1.00	1.00	0.03	0.8529
**s (PDC mean anti-platelet agents) male**		2.43	2.43	10.20	0.0000***
**s (PDC mean anti-platelet agents) female**		1.00	1.00	9.76	0.0018**
**s (PDC standard deviation ACE inhibitors) male**		2.93	2.93	4.86	0.0023**
**s (PDC standard deviation ACE inhibitors) female**		2.87	2.87	6.88	0.0002***
**s (PDC standard deviation β-blockers) male**		4.70	4.70	17.23	0.0000***
**s (PDC standard deviation β-blockers) female**		1.00	1.00	3.78	0.0520
**s (PDC standard deviation statins) male**		1.18	1.18	1.06	0.3681
**s (PDC standard deviation statins) female**		3.59	3.59	3.45	0.0136*
**s (PDC standard deviation anti-platelet agents) male**		3.42	3.42	8.22	0.0000***
**s (PDC standard deviation anti-platelet agents) female**		2.93	2.93	9.49	0.0000***

R-sq. (adj.) = 0.338

*Abbreviations*: *AMI* (Acute Myocardial infarction), *BIMD 2010* (Bavarian Index of Multiple Deprivation, year 2010), *BMI* (Body Mass Index), *DMP* (Disease Management Program), *HMG* (Hierarchical Morbidity Group), *NYHA* (New York Hear Association), *PDC* (Proportion of days covered)

Significane Levels:* *p* < 0.05 ** *p* <0.01 *** *p* < 0.001

## Discussion

### Main results

This is the first study analyzing the influence of PDC rates for guideline-recommended medication after AMI on health care expenditures with longitudinal real-world data. It seems that the absolute mean PDC-rate (inter-individual effect) has only minimal influence, while a deviation from this mean (intra-individual effect) has a large impact on health care expenditures. These results were quite robust in sensitivity analyses. Two different effects may partly explain this phenomenon. First, health care expenditures in the first year after AMI were much higher than in consecutive years, and the deviation from the mean PDC rate in the first year is positive most of the time. Second, a positive deviation in PDC rates might be the reason for reverse causation, as it could be an indicator of worsening of the health status of a patient, leading to higher adherence to guideline-recommended medication. We adjusted for both effects by including the year after AMI and a time-varying comorbidity index into the regression analysis. Even after adjustment for possible confounders, we observed almost no inter-individual effect but a considerable intra-individual effect. This means that the absolute adherence rates across all individuals are associated with lower changes in health care expenditures than the intra-individual effect (a change in the individual’s adherence over time). This might imply that factors associated with variation of adherence at an individual level, e.g., a change in an individual doctor’s prescribing habits or changes in an individual’s consumption of guideline-based medication should be avoided in practice for patients after an AMI.

Previously published literature from clinical trials led to the assumption that differences between sexes might exist regarding the effectiveness of β-blockers [38–40], and ACE inhibitors [41–43]. Interestingly, mean PDC rates as well as deviations from mean PDC rates for both men and women were significant in anti-platelet agents. For statins, the intra-individual effect (deviation from the mean PDC rate) became significant only in female. Significant differences between deviations from the mean were seen in ACE inhibitors in females, and β-blockers in males, which is in line with the findings of the clinical trials. For ACE inhibitors a deviation from the mean PDC-rate seems to be connected to a higher increase in costs in females compared with males. With respect to β-blockers, an increasing negative deviation is associated with increasing costs, although an increasing positive deviation is associated with increasing costs; this could be found in both sexes but was more pronounced in males.

Overall, observed mean PDC rates were lower than expected, given a threshold for adherence of 80% in most publications [50–54]. Only the mean for statins was above 80% in men. Especially for anti-platelet agents, PDC rates were low in both sexes, ranging from 46.42% in males in the first year after AMI to 25.41% in females in the third year. A possible explanation for the low PDC rates for anti-platelet agents could be that Aspirin 100 mg, as recommended by the guidelines, is available as an over the counter medication, which is why its use might not be fully reflected in the data from statutory health insurance. The quite moderate declines from years 1 to 3 in β-blockers are interesting, as the guidelines [9, 10] recommend β-blockers after AMI only for up to 2 years.

### Comparison with the literature

Only three studies [28–30] have been published so far measuring the influence on health care expenditures of adherence to one [28, 29] or two [30] guideline-recommended medications after AMI.

In a retrospective claims data analysis of a large US health insurer, Bansilal et al. [30] measured the influence of statins and ACE inhibitor adherence on hospitalization costs in a follow-up period of up to 3 years; this was measured using PDC rates (> 80% fully adherent; 40–79% partially adherent; and < 40% non-adherent). Full adherence to statins and ACE inhibitors was associated with reduced per-patient annual hospitalization costs for AMI and for revascularization procedures compared with partial and non-adherence.

Sun et al. [28] analyzed the influence of costs of adherence in a large US national pharmacy-benefit database, which was measured by the medication possession ratio (MPR) (> 80% fully adherent; 40–79% partially adherent; and < 40% non-adherent), in a 1-year follow-up period, to renin–angiotensin system agents (ACE inhibitors or angiotensin receptor blockers). They found that partially adherent and fully adherent groups had significantly lower cardiovascular-related and total health care costs than the non-adherent group.

Summaria et al. [29] measured the influence of statin adherence on health care expenditures in an Italian retrospective observational study of an administrative database (not further specified), in a follow-up period of up to 3 years, using MPR (> 80%; 50–79%, 25–50, < 25%). They found that mean health care expenditures increased from the non-adherent to the fully adherent group.

By focusing on PDCs [30] and MPRs [28, 29] previous studies have used measures of adherence that were quite similar, yet with varying adherence thresholds, except for the fully adherent group (which was > 80% in all studies). The results of the two US studies [28, 30] indicate cost savings, whereas the only European (Italian) study [29] reported the highest health care expenditures in the fully adherent group.

Our results are not directly comparable, as we used a longitudinal approach considering all four guideline-recommended medications simultaneously. In addition, we chose an inter- (mean PDC rate over the complete period) and intra-individual (deviation of the mean PDC rate of a person in the observed year) approach to measure the influence of adherence on health care expenditures. Nevertheless, the findings of our analysis are to some degree in line with earlier findings, as we found no influence of statins but a significant negative influence of higher PDC rates in ACE inhibitors on health care expenditures in women.

So far, there have been no previous observational studies based on German data that would allow comparison of our results regarding health care expenditures. Comparison is possible only with respect to adherence to guideline-recommended medication and its development over time [18, 25, 55]. In this regard, our findings are quite similar to their observations.

In the Cologne Infarction Model (KIM), Reuter and colleagues [25] measured self-reported adherence of 610 consecutive patients with ST-elevation myocardial infarction (STEMI) treated by primary percutaneous coronary intervention (PCI) at hospital discharge and after a median follow-up period of 36 months. Respective proportions of adherence for ASS, statins, β-blockers, ACE inhibitors, or angiotensin-receptor blockers at hospital discharge were between 90.8 and 97.6% and at follow-up between 79.2 and 90.8%.

Amann et al. [55] measured self-reported adherence in 1667 AMI patients from the MONICA/KORA cohort in a survey at hospital discharge and at a mean follow-up time of 6.1 years. The proportion of patients taking anti-platelet agents, β-blockers, statins, and renin–angiotensin–aldosterone system blockers was between 83.6 and 97.5% at hospital discharge and between 79.3 and 90.9% at follow-up.

Mangiapane et al. [18] analyzed prescriptions in a sample of 30,028 AMI patients insured at Techniker Krankenkasse. They found that prescription rates declined from 82% in β-blockers, 73% in statins, 69% in ACE inhibitors, and 66% in platelet aggregation inhibitors at hospital discharge to 36% in β-blockers, 17% in statins, 31% in ACE inhibitors, and 10% in platelet aggregation inhibitors after a follow-up of 5 years. Although different methods for determining adherence were used (self-reported adherence [25, 55], treatment persistence [18], and yearly PDC rates in our study), the findings indicate a general decline in guideline-recommended medication intake over time after AMI. The decline appears to be even more pronounced when the data basis is claims data rather than self-reported data.

### Limitations

Some potential limitations of this study should be considered while interpreting the results.

For β-blockers, national guidelines [32] recommend intake only for 1 up to 2 years after the AMI; we measured the mean PDC rate over the complete 3-year follow-up period, which might underestimate the positive impact of adherence to β-blockers.

Pharmacy dispensing data were used as a measure of PDC rates, which does not allow definite judgment as to whether patients had actually taken the paid for and collected medication. However, pharmacy refill records have been found to be highly correlated with electronic adherence monitoring, and the act of refilling a medication has been argued to reflect the patient’s active decision to continue with therapy [56]. Furthermore, the number of days that needed to be covered with prescribed medication was reduced by the number of hospital days in the follow-up period because medication was presumably provided by the hospital during hospitalization. This means that a higher percentage of time spent in hospital, which increases costs, also increases the mean PDC rate and positive deviation from the mean PDC rate. In the first sensitivity analysis, we excluded a relatively small number of patients who were hospitalized for more than 50% of the days observed. These patients were mainly high-cost patients with high PDC rates, owing to the high percentage of time spent in hospital, where they received guideline-recommended medication per definition. Excluding these patients allows us to invalidate the impression that a higher PDC rate was associated with higher health care expenditures resulting from reversed causation.

Adherence to anti-platelet agents may be underestimated, as Aspirin 100 mg has a co-payment of 100% and is available over the counter. Although physicians can still prescribe aspirin after AMI, its removal from reimbursement had a clear effect on prescription incidence, which dropped from 72% in 2003 to 57% in 2004 [18]. An ongoing prescription of anti-platelet agents might identify high-risk patients, as the physician makes sure, by the inclusion of Aspirin 100 mg on the prescription, that the patient has no need to order it in the pharmacy. Therefore, our finding that a higher PDC rate in anti-platelet agents causes higher health care expenditures should be interpreted with caution.

We did not exclude those who were never prescribed any of the four medications as other studies measuring adherence or persistence did [29, 30]. We wanted to measure adherence to guideline-recommended medication, which is the same for every patient after AMI, except for contraindications, which we could not capture in a retrospective claims data analysis. Therefore, the findings might not be directly comparable.

Our study population was enrolled in the DMP CAD at index AMI, which is voluntary, and therefore we could not exclude a self-selection effect of patients leading to an overestimation of PDC rates. However, the DMP CAD might include the more severe cases, as diagnosed CAD, which is an inclusion criterion for the DMP, existed before AMI.

Finally, as individual socio economic status is usually not sufficiently reflected in routine data, we incorporated an area-based deprivation index for Bavaria (BIMD 2010) as a proxy. Nevertheless, this procedure is a standard approach in corresponding studies utilizing claims or register data, and the index used is a well-established and recognized tool to address such limitations [47].

Aside from these aspects, to the best of our knowledge, this is the first study considering the influence of all four guideline-recommended medications on health care expenditures, which likely gives a more realistic picture of the effectiveness of adherence to guideline-recommended medications after AMI because positive correlation of adherence to other guideline-recommended medications is accounted for [55]. If this effect is not considered, the positive impact of one type of medication might be overestimated, as the positive impact of adherence to another type of medication is attributed to the medication under scrutiny.

Additionally, our sample size was large enough to stratify the analysis for sex, as there is evidence that there are differences in the effectiveness of ACE inhibitors and β-blockers between females and males. To what extent these differences influence health care expenditures has not been investigated to date.

Furthermore, the GAMM incorporates a longitudinal design, which seems to be more appropriate than a cross-sectional design, as it also controls for individual changes over time.

In addition, a relatively long period of 4 years was available for every patient, which means that information from the year before AMI could be considered in the analyses, such as medication stocks that patients had before the AMI, leading to a more realistic estimate of the PDC rate.

## Conclusion

This is the first study to consider the influence of medications after AMI on health care expenditures for a population of DMP patients reflected by routine care in Germany. Unlike previous studies, we considered adherence regarding all four guideline-recommended medications simultaneously. A longitudinal stratified design allowed the capture of variation in adherence over time and sex-specific differences. Using a GAMM, we were able to take into account inter-individual and intra-individual effects and, thereby, allow for a more complete analysis. The overall low and (over time) declining PDC rates for all guideline-recommended medications found in this study may be attributable to using real-world data from a large statutory health insurance fund rather than self-reported data. Although we cannot confirm the results of clinical studies, which mainly found cost savings for adherence after AMI, we found that deviation in the PDC means (the intra-personal effect) in either direction seemed to have a greater impact on health care expenditures than the mean PDC rate (inter-personal effect). It is possible that, for the patients who would presumably have been excluded from clinical trials, effectiveness is not given in the same way as shown in clinical trials, leading to higher costs despite being adherent. In the same way, the findings of other observational studies [28–30] do not consistently report reductions in health care expenditures. Therefore, it seems to be necessary for further analyses of real-world data, such as registries and claims data, to be conducted to unveil cost saving potentials related to guideline-recommended adherence after AMI and, in particular, factors influencing individual-level variation in adherence.

## Supplementary Information

**Additional file 1.**

**Additional file 2.**

**Additional file 3.**

## Data Availability

The datasets generated and/or analyzed during the current study are not publicly available due to §75 SGB X but are available from the corresponding author on reasonable request.

## Abbreviations

ACE: Angiotensin-converting enzyme
AMI: Acute myocardial infarction
ANOVA: Analysis of variance
ATC: Anatomical therapeutic chemical
BIMD 2010: Bavarian Index of Multiple Deprivation of 2010
BMI: Body Mass Index
CAD: Coronary artery disease
COPD: Chronic obstructive pulmonary disease
CVD: Cardiovascular disease
DDD: Defined daily dose
DMP: Disease Management Program
GAMM: Generalized additive mixed model
HMG: Hierarchical morbidity group
KIM: Cologne Infarction Model
MPR: Medication possession ratio
NYHA: New York Heart Association classification
PCI: Percutaneous coronary intervention
PDC: Proportion of days covered
STEMI: ST-elevation myocardial infarction
WIdO: The scientific institute of the AOK

## References

[1] Moran AE, Forouzanfar MH, Roth GA, Mensah GA, Ezzati M, Flaxman A (2014). The global burden of ischemic heart disease in 1990 and 2010: the global burden of disease 2010 study. Circulation..

[2] Heidenreich PA, Trogdon JG, Khavjou OA, Butler J, Dracup K, Ezekowitz MD (2011). Forecasting the future of cardiovascular disease in the United States: a policy statement from the American Heart Association. Circulation..

[3] Likosky DS, Zhou W, Malenka DJ, Borden WB, Nallamothu BK, Skinner JS (2013). Growth in medicare expenditures for patients with acute myocardial infarction: a comparison of 1998 through 1999 and 2008. JAMA Intern Med.

[4] Go AS, Mozaffarian D, Roger VL, Benjamin EJ, Berry JD, Blaha MJ (2014). Executive summary: heart disease and stroke statistics--2014 update: a report from the American Heart Association. Circulation..

[5] Gesundheitsberichterstattung des Bundes. Sterbefälle, Sterbeziffern (je 100.000 Einwohner, alterstandardisiert). https://www.gbebund.de/gbe/pkg_isgbe5.prc_menu_olap?p_uid=gast&p_aid=82572887&p_sprache=D&p_help=2&p_indnr=6&p_indsp=&p_ityp=H&p_fid=. Accessed 30 Nov 2020.

[6] Reinhold T, Lindig C, Willich SN, Brüggenjürgen B (2011). The costs of myocardial infarction—a longitudinal analysis using data from a large German health insurance company. J Public Health.

[7] Russell MW, Huse DM, Drowns S, Hamel EC, Hartz SC (1998). Direct medical costs of coronary artery disease in the United States. Am J Cardiol.

[8] Lacey L, Tabberer M (2005). Economic burden of post-acute myocardial infarction heart failure in the United Kingdom. Eur J Heart Fail.

[9] Van de Werf F, Bax J, Betriu A, Blomstrom-Lundqvist C, Crea F, Falk V (2009). ESC guidelines on management of acute myocardial infarction in patients presenting with persistent ST-segment elevation. Revista espanola de cardiologia.

[10] Gandjour A, Stock S (2007). A national hypertension treatment program in Germany and its estimated impact on costs, life expectancy, and cost-effectiveness. Health Policy (Amsterdam, Netherlands).

[11] Stark R, Kirchberger I, Hunger M, Heier M, Leidl R, von Scheidt W (2014). Improving care of post-infarct patients: effects of disease management programmes and care according to international guidelines. Clin Res Cardiol.

[12] Zeymer U (2007). Secondary prevention in outpatients with coronary artery disease. Adherence with recommendations within 4 weeks after hospital discharge. Deutsche medizinische Wochenschrift (1946).

[13] Mihaylova B, Emberson J, Blackwell L, Keech A, Simes J, Barnes EH (2012). The effects of lowering LDL cholesterol with statin therapy in people at low risk of vascular disease: meta-analysis of individual data from 27 randomised trials. Lancet.

[14] Law MR, Morris JK, Wald NJ (2009). Use of blood pressure lowering drugs in the prevention of cardiovascular disease: meta-analysis of 147 randomised trials in the context of expectations from prospective epidemiological studies. BMJ.

[15] Baigent C, Blackwell L, Collins R, Emberson J, Godwin J, Peto R (2009). Aspirin in the primary and secondary prevention of vascular disease: collaborative meta-analysis of individual participant data from randomised trials. Lancet.

[16] Boger GI, Hoopmann M, Busse R, Budinger M, Welte T, Boger RH (2003). Drug therapy of coronary heart disease--are therapeutic guidelines being paid attention to?. Zeitschrift fur Kardiologie.

[17] Frilling B, Schiele R, Gitt AK, Zahn R, Schneider S, Glunz HG (2004). Too little aspirin for secondary prevention after acute myocardial infarction in patients at high risk for cardiovascular events: results from the MITRA study. Am Heart J.

[18] Mangiapane S, Busse R (2011). Prescription prevalence and continuing medication use for secondary prevention after myocardial infarction: the reality of care revealed by claims data analysis. Deutsches Arzteblatt international.

[19] van der Elst ME, Bouvy ML, de Blaey CJ, de Boer A (2005). Preventive drug use in patients with a history of nonfatal myocardial infarction during 12-year follow-up in the Netherlands: a retrospective analysis. Clin Ther.

[20] Bischoff B, Silber S, Richartz BM, Pieper L, Klotsche J, Wittchen HU (2006). Inadequate medical treatment of patients with coronary artery disease by primary care physicians in Germany. Clin Res Cardiol.

[21] Gasse C, Jacobsen J, Larsen AC, Schmidt EB, Johannesen NL, Videbaek J (2008). Secondary medical prevention among Danish patients hospitalised with either peripheral arterial disease or myocardial infarction. Eur J Vasc Endovasc Surg.

[22] Senst BL, Achusim LE, Genest RP, Cosentino LA, Ford CC, Little JA (2001). Practical approach to determining costs and frequency of adverse drug events in a health care network. Am J Health Syst Pharm.

[23] Rodgers PT, Ruffin DM (1998). Medication nonadherence: part II--A pilot study in patients with congestive heart failure. Manag Care Interface.

[24] Benner JS, Glynn RJ, Mogun H, Neumann PJ, Weinstein MC, Avorn J (2002). Long-term persistence in use of statin therapy in elderly patients. Jama..

[25] Reuter H, Markhof A, Scholz S, Wegmann C, Seck C, Adler C (2015). Long-term medication adherence in patients with ST-elevation myocardial infarction and primary percutaneous coronary intervention. Eur J Prev Cardiol.

[26] Osterberg L, Blaschke T (2005). Adherence to medication. N Engl J Med.

[27] Cutler DM, Everett W (2010). Thinking outside the pillbox--medication adherence as a priority for health care reform. N Engl J Med.

[28] Sun SX, Ye X, Lee KY, Dupclay L, Plauschinat C (2008). Retrospective claims database analysis to determine relationship between renin-angiotensin system agents, rehospitalization, and health care costs in patients with heart failure or myocardial infarction. Clin Ther.

[29] Summaria F, Ciaralli F, Mustilli M, Sette A, Lanzillo C, Vasselli L (2013). Pharmacoeconomic impact evaluation of statin adherence in high-risk unselected post myocardial infarction population: an administrative database-guided analysis. Medical Archives (Sarajevo, Bosnia and Herzegovina).

[30] Bansilal S, Castellano JM, Garrido E, Wei HG, Freeman A, Spettell C (2016). Assessing the impact of medication adherence on long-term cardiovascular outcomes. J Am Coll Cardiol.

[31] Schafer I, Kuver C, Gedrose B, Hoffmann F, Russ-Thiel B, Brose HP (2010). The disease management program for type 2 diabetes in Germany enhances process quality of diabetes care - a follow-up survey of patient's experiences. BMC Health Serv Res.

[32] Serxner S, Baker K, Gold D (2006). Guidelines for analysis of economic return from health management programs. Am J Health Promotion.

[33] Consumer prices [Internet]. 2018. Available from: https://www.oecd-ilibrary.org/content/data/0f2e8000-en. Accessed 30 Nov 2020.

[34] Berger JS, Roncaglioni MC, Avanzini F, Pangrazzi I, Tognoni G, Brown DL (2006). Aspirin for the primary prevention of cardiovascular events in women and men: a sex-specific meta-analysis of randomized controlled trials. Jama..

[35] Antithrombotic Trialists' Collaboration. Collaborative meta-analysis of randomised trials of antiplatelet therapy for prevention of death, myocardial infarction, and stroke in high risk patients. BMJ. 2002;324.7329(2002):71–86. 10.1136/bmj.324.7329.71 PMC64503 11786451

[36] LaRosa JC, He J, Vupputuri S (1999). Effect of statins on risk of coronary disease: a meta-analysis of randomized controlled trials. Jama..

[37] Cheung BM, Lauder IJ, Lau CP, Kumana CR (2004). Meta-analysis of large randomized controlled trials to evaluate the impact of statins on cardiovascular outcomes. Br J Clin Pharmacol.

[38] Labbe L, Sirois C, Pilote S, Arseneault M, Robitaille NM, Turgeon J (2000). Effect of gender, sex hormones, time variables and physiological urinary pH on apparent CYP2D6 activity as assessed by metabolic ratios of marker substrates. Pharmacogenetics..

[39] Luzier AB, Killian A, Wilton JH, Wilson MF, Forrest A, Kazierad DJ (1999). Gender-related effects on metoprolol pharmacokinetics and pharmacodynamics in healthy volunteers. Clin Pharmacol Ther.

[40] Jochmann N, Stangl K, Garbe E, Baumann G, Stangl V (2005). Female-specific aspects in the pharmacotherapy of chronic cardiovascular diseases. Eur Heart J.

[41] Shekelle PG, Rich MW, Morton SC, Atkinson CSW, Tu W, Maglione M (2003). Efficacy of angiotensin-converting enzyme inhibitors and beta-blockers in the management of left ventricular systolic dysfunction according to race, gender, and diabetic status: a meta-analysis of major clinical trials. J Am Coll Cardiol.

[42] Fox KM (2003). Efficacy of perindopril in reduction of cardiovascular events among patients with stable coronary artery disease: randomised, double-blind, placebo-controlled, multicentre trial (the EUROPA study). Lancet.

[43] Wing LM, Reid CM, Ryan P, Beilin LJ, Brown MA, Jennings GL (2003). A comparison of outcomes with angiotensin-converting--enzyme inhibitors and diuretics for hypertension in the elderly. N Engl J Med.

[44] Hedeker D (2004). An introduction to growth modeling. The Sage handbook of quantitative methodology for the social sciences.

[45] Wood S, Scheipl F, Wood MS (2017). Package ‘gamm4’. Am Stat.

[46] Wood S, Scheipl F. gamm4: Generalized additive mixed models using mgcv and lme4. 2014. http://CRAN.R-project.org/package=gamm4. Accessed 20 Nov 2020.

[47] Maier W, Fairburn J, Mielck A (2012). Regional deprivation and mortality in Bavaria. Development of a community-based index of multiple deprivation. Gesundheitswesen..

[48] Bauer H, Maier W. GIMD 2010–Ein Update des‚ German Index of Multiple Deprivation. Berichte des Helmholtz Zentrums München. 2018.

[49] Noble M, Wright G, Smith G, Dibben C (2006). Measuring multiple deprivation at the small-area level. Environ Plan A.

[50] Hamood H, Hamood R, Green MS, Almog R (2016). Determinants of adherence to evidence-based therapy after acute myocardial infarction. Eur J Prev Cardiol.

[51] Ho PM, Magid DJ, Masoudi FA, McClure DL, Rumsfeld JS (2006). Adherence to cardioprotective medications and mortality among patients with diabetes and ischemic heart disease. BMC Cardiovasc Disord.

[52] Chapman RH, Benner JS, Petrilla AA, Tierce JC, Collins SR, Battleman DS (2005). Predictors of adherence with antihypertensive and lipid-lowering therapy. Arch Intern Med.

[53] Steiner JF, Prochazka AV (1997). The assessment of refill compliance using pharmacy records: methods, validity, and applications. J Clin Epidemiol.

[54] Karve S, Cleves MA, Helm M, Hudson TJ, West DS, Martin BC (2008). An empirical basis for standardizing adherence measures derived from administrative claims data among diabetic patients. Med Care.

[55] Amann U, Kirchberger I, Heier M, Thilo C, Kuch B, Meisinger C (2018). Medication use in long-term survivors from the MONICA/KORA myocardial infarction registry. Eur J Internal Med.

[56] Krousel-Wood M, Thomas S, Muntner P, Morisky D (2004). Medication adherence: a key factor in achieving blood pressure control and good clinical outcomes in hypertensive patients. Curr Opin Cardiol.

