# Supplementary material for: Effects of adherence to pharmacological secondary prevention after acute myocardial infarction on health care costs – an analysis of real-world data
Source: BMC Health Serv Res. 2020 Dec 20;20:1145. doi: 10.1186/s12913-020-05946-4 (PMC7751107; doi:10.1186/s12913-020-05946-4)
Supplement: Supplementary file 2 — Additional file 2. [file 12913_2020_5946_MOESM2_ESM.docx]

**Online figure 1: Base Case – Influence on ambulatory costs**


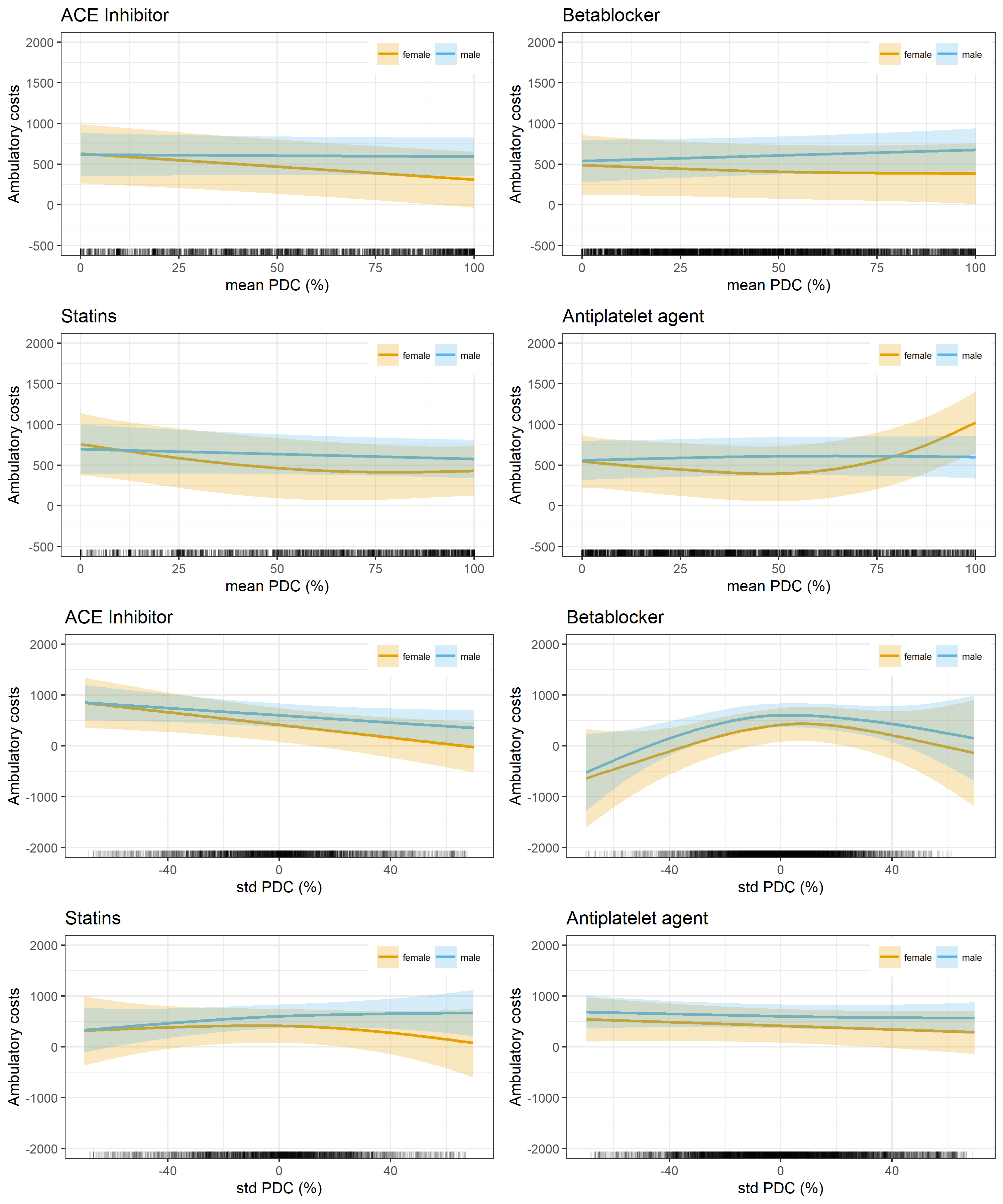


**Online figure 2: Base Case – Influence on medication costs**


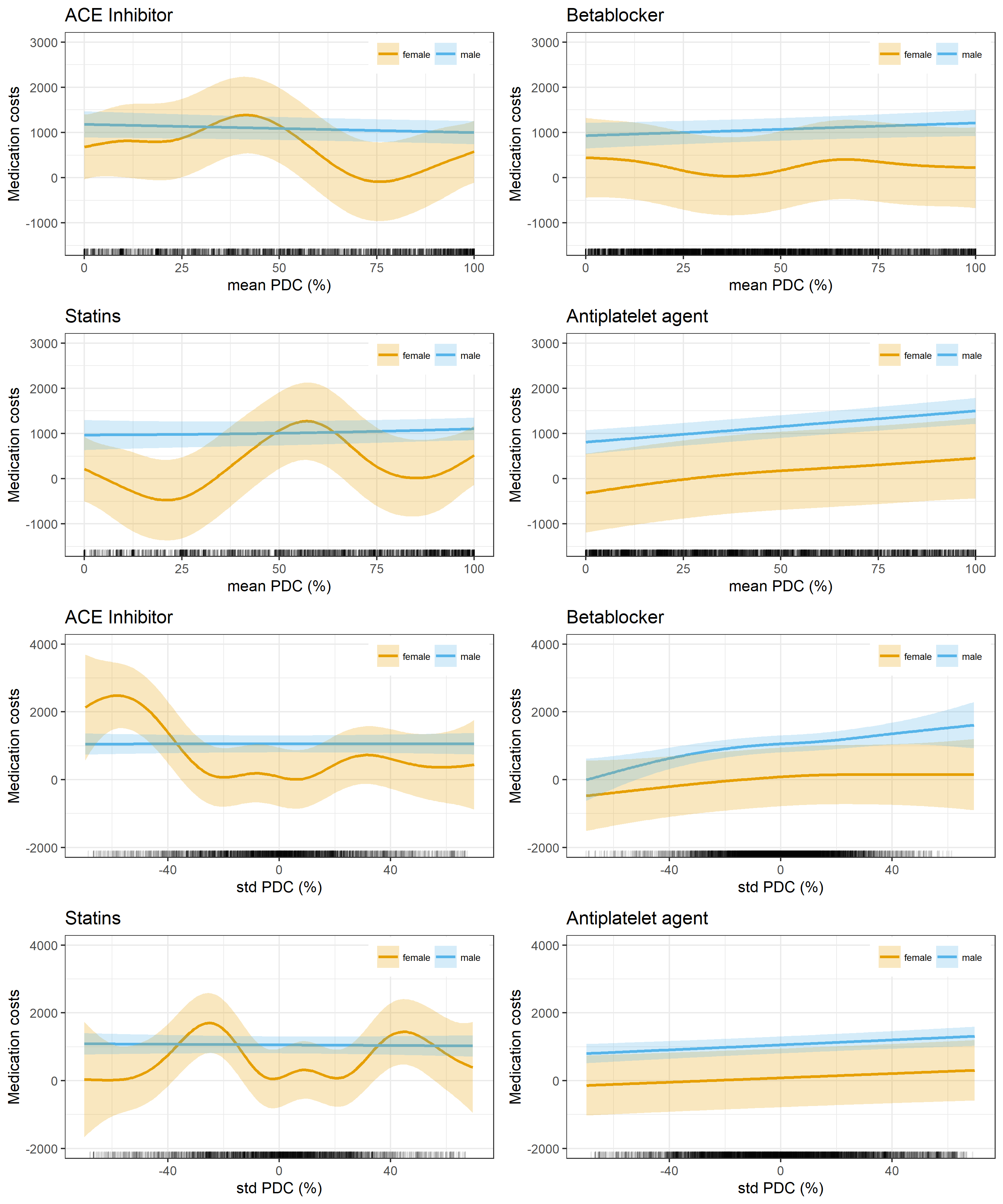


**Online figure 3: Base Case – Influence on hospitalization costs**


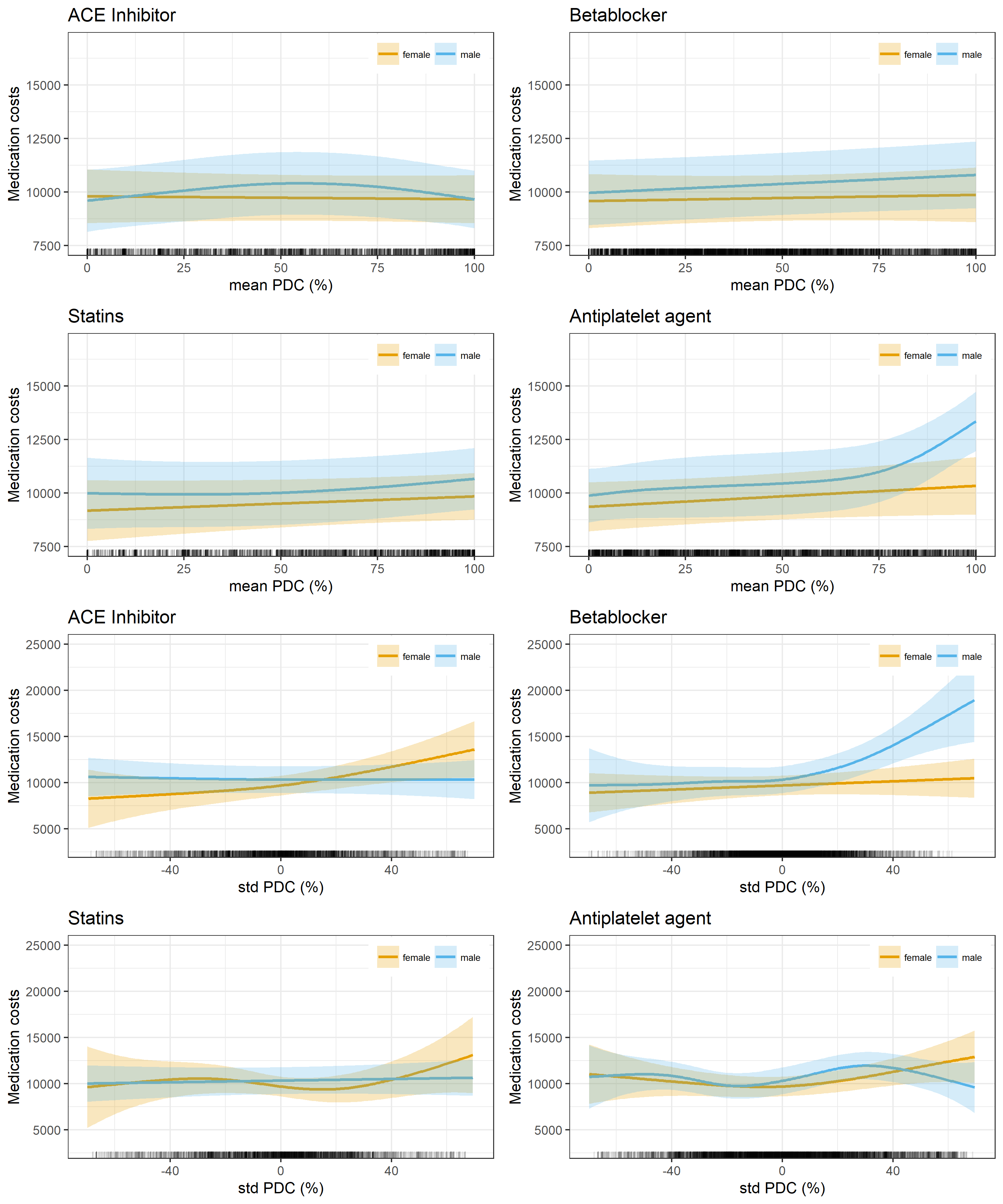


**Online figure 4: Base Case – Influence on rehabilitation costs**


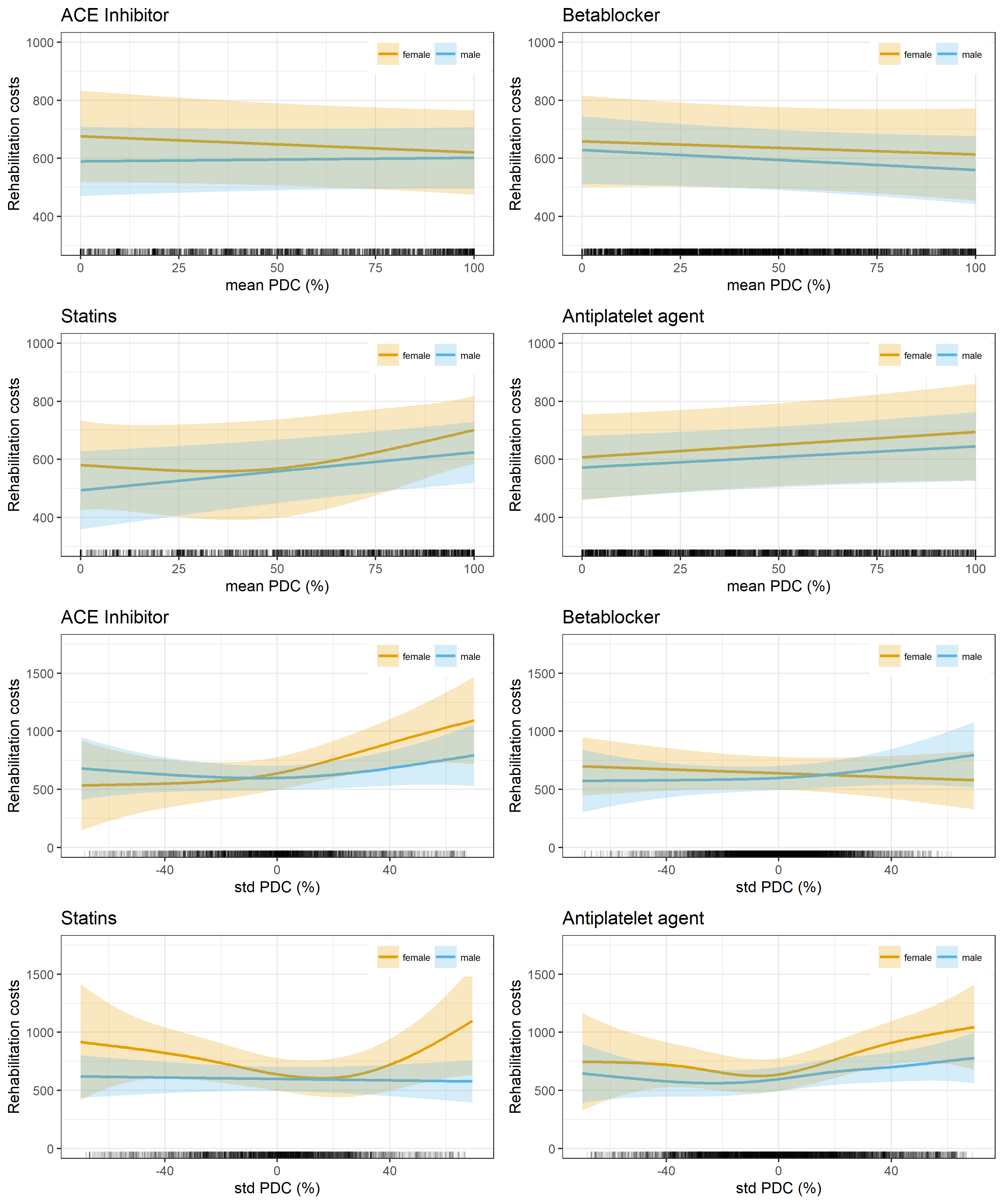


**Online figure 5: Base Case – Influence on remedy and aids costs**

**
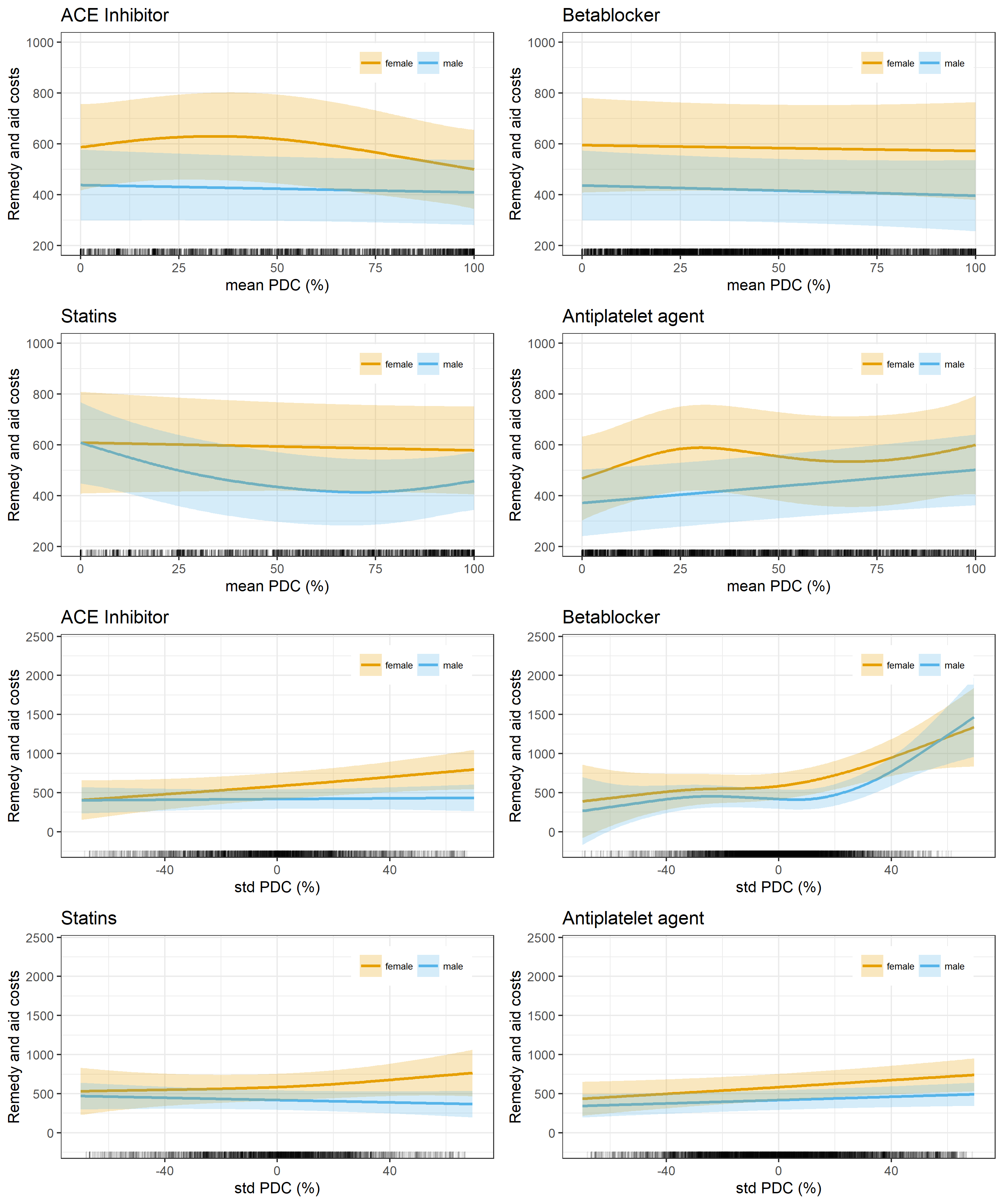
**
