# Supplementary material for: Effects of adherence to pharmacological secondary prevention after acute myocardial infarction on health care costs – an analysis of real-world data
Source: BMC Health Serv Res. 2020 Dec 20;20:1145. doi: 10.1186/s12913-020-05946-4 (PMC7751107; doi:10.1186/s12913-020-05946-4)
Supplement: Supplementary file 3 — Additional file 3. [file 12913_2020_5946_MOESM3_ESM.docx]

Online table 1: Base Case – Influence of PDC rates on ambulatory costs

|  |  | **Estimate** | **Std. Error** | **t value** | **Pr(>\|t\|)** |
| --- | --- | --- | --- | --- | --- |
| **(Intercept)** |  | -1297.79 | 365.93 | -3.55 | 0.0004*** |
| **Age** | **55 < 65** | -127.82 | 144.61 | -0.88 | 0.3768 |
|  | **65 < 75** | -119.81 | 136.28 | -0.88 | 0.3793 |
|  | **≥ 75** | -98.58 | 137.35 | -0.72 | 0.4729 |
| **Gender** | **female** | 34.15 | 66.55 | 0.51 | 0.6078 |
| **BMI** | **underweight** | -197.21 | 371.36 | -0.53 | 0.5954 |
|  | **overweight** | -19.06 | 75.14 | -0.25 | 0.7998 |
|  | **obese** | -32.94 | 82.71 | -0.40 | 0.6904 |
| **BIMD 2010 (Q1 least deprived, Q5 most deprived)** | **Q2** | 126.54 | 92.11 | 1.37 | 0.1695 |
|  | **Q3** | 146.52 | 98.59 | 1.49 | 0.1373 |
|  | **Q4** | -7.50 | 97.54 | -0.08 | 0.9387 |
|  | **Q5** | 47.42 | 92.56 | 0.51 | 0.6084 |
| **Smoker** | **yes** | 77.11 | 93.48 | 0.82 | 0.4095 |
| **NYHA** | **1** | -28.69 | 169.33 | -0.17 | 0.8655 |
|  | **2** | -95.95 | 99.42 | -0.97 | 0.3345 |
|  | **3** | -121.24 | 88.95 | -1.36 | 0.1729 |
|  | **4** | -72.22 | 93.34 | -0.77 | 0.4391 |
| **DMP COPD** | **yes** | -10.19 | 108.40 | -0.09 | 0.9251 |
| **DMP asthma** | **yes** | 111.82 | 186.69 | 0.60 | 0.5492 |
| **DMP diabetes type 1** | **yes** | 574.51 | 513.88 | 1.12 | 0.2636 |
| **DMP diabetes type 2** | **yes** | 67.30 | 60.93 | 1.10 | 0.2694 |
| **deceased** | **yes** | 193.65 | 173.36 | 1.12 | 0.2640 |
| **HMG assignments per month** |  | 0.31 | 0.05 | 6.59 | 0.0000*** |
| **Year after AMI** |  | -59.29 | 29.48 | -2.01 | 0.0444* |
| **days insured** |  | 3.97 | 0.88 | 4.49 | 0.0000*** |
| **Angina pectoris** |  | 65.36 | 54.88 | 1.19 | 0.2337 |
| **Peripheral vascular disease** |  | 58.23 | 74.31 | 0.78 | 0.4333 |
| **Dyslipidemia** |  | 126.18 | 71.38 | 1.77 | 0.0771 |
| **Congestive heart failure** |  | -96.99 | 68.02 | -1.43 | 0.1540 |
| **Hypertension** |  | -2.67 | 110.00 | -0.02 | 0.9806 |
| **Dialysis** |  | 15712.01 | 195.48 | 80.38 | 0.0000*** |
|  |  | **edf** | **Ref.df** | **F** | **p-value** |
| **s(PDC mean ACE inhibitors) male** |  | 1.00 | 1.00 | 0.07 | 0.7899 |
| **s(PDC mean ACE inhibitors) female** |  | 1.00 | 1.00 | 6.54 | 0.0105* |
| **s(PDC mean β-blockers) male** |  | 1.00 | 1.00 | 1.24 | 0.2662 |
| **s(PDC mean β-blockers) female** |  | 1.21 | 1.21 | 0.21 | 0.6096 |
| **s(PDC mean statins) male** |  | 1.00 | 1.00 | 1.01 | 0.3150 |
| **s(PDC mean statins) female** |  | 1.57 | 1.57 | 4.33 | 0.0689 |
| **s(PDC mean anti-platelet agents) male** |  | 1.30 | 1.30 | 0.11 | 0.6910 |
| **s(PDC mean anti-platelet agents) female** |  | 2.62 | 2.62 | 4.20 | 0.0053** |
| **s(PDC standard deviation ACE inhibitors) male** |  | 1.00 | 1.00 | 3.62 | 0.0570 |
| **s(PDC standard deviation ACE inhibitors) female** |  | 1.00 | 1.00 | 5.49 | 0.0191* |
| **s(PDC standard deviation β-blockers) male** |  | 2.94 | 2.94 | 4.26 | 0.0044** |
| **s(PDC standard deviation β-blockers) female** |  | 2.54 | 2.54 | 3.74 | 0.0210* |
| **s(PDC standard deviation statins) male** |  | 1.42 | 1.42 | 0.83 | 0.2761 |
| **s(PDC standard deviation statins) female** |  | 1.57 | 1.57 | 0.66 | 0.6015 |
| **s(PDC standard deviation anti-platelet agents) male** |  | 1.18 | 1.18 | 0.54 | 0.5564 |
| **s(PDC standard deviation anti-platelet agents) female** |  | 1.00 | 1.00 | 0.81 | 0.3677 |
| R-sq. (adj.) = 0.587 | | | | | |
| **Abbreviations: AMI (Acute Myocardial infarction), BIMD2010 (Bavarian Index of Multiple Deprivation, year 2010), BMI (Body Mass Index), DMP (Disease Management Program), HMG (Hierarchical Morbidity Group), NYHA (New York Hear Association), PDC (Proportion of days covered)** | | | | | |

Online table 2: Base Case - Influence of PDC rates on medication costs

| **N = 9,287** |  | **Estimate** | **Std. Error** | **t value** | **Pr(>\|t\|)** |
| --- | --- | --- | --- | --- | --- |
| **(Intercept)** |  | -387.80 | 336.14 | -1.15 | 0.2487 |
| **Age** | **55 < 65** | -103.37 | 152.35 | -0.68 | 0.4975 |
|  | **65 < 75** | -151.16 | 148.68 | -1.02 | 0.3093 |
|  | **≥ 75** | -274.53 | 150.27 | -1.83 | 0.0677** |
| **Gender** | **female** | 210.33 | 77.43 | 2.72 | 0.0066 |
| **BMI** | **underweight** | 146.83 | 379.06 | 0.39 | 0.6985 |
|  | **overweight** | 114.20 | 75.51 | 1.51 | 0.1305 |
|  | **obese** | 74.66 | 86.89 | 0.86 | 0.3903 |
| **BIMD 2010 (Q1 least deprived,Q5 most deprived)** | **Q2** | 26.77 | 107.38 | 0.25 | 0.8032 |
|  | **Q3** | 121.78 | 114.37 | 1.06 | 0.2870 |
|  | **Q4** | -59.26 | 113.48 | -0.52 | 0.6015 |
|  | **Q5** | 6.66 | 107.57 | 0.06 | 0.9506 |
| **Smoker** | **yes** | -88.64 | 97.15 | -0.91 | 0.3616 |
| **NYHA** | **1** | 124.96 | 178.34 | 0.70 | 0.4835 |
|  | **2** | -73.29 | 101.68 | -0.72 | 0.4711 |
|  | **3** | 76.19 | 90.05 | 0.85 | 0.3975 |
|  | **4** | -11.67 | 94.49 | -0.12 | 0.9017 |
| **DMP COPD** | **yes** | 466.56 | 116.19 | 4.02 | 0.0001*** |
| **DMP asthma** | **yes** | 43.16 | 210.79 | 0.20 | 0.8378 |
| **DMP diabetes type 1** | **yes** | 2944.09 | 549.13 | 5.36 | 0.0000*** |
| **DMP diabetes type 2** | **yes** | 246.09 | 66.82 | 3.68 | 0.0002*** |
| **deceased** | **yes** | 57.99 | 151.03 | 0.38 | 0.7010 |
| **HMG assignments per month** |  | 0.31 | 0.04 | 7.53 | 0.0000*** |
| **Year after AMI** |  | -243.40 | 22.28 | -10.93 | 0.0000*** |
| **days insured** |  | 3.75 | 0.78 | 4.81 | 0.0000*** |
| **Angina pectoris** |  | 38.47 | 48.50 | 0.79 | 0.4277 |
| **Peripheral vascular disease** |  | 169.80 | 61.19 | 2.78 | 0.0055** |
| **Dyslipidemia** |  | 65.10 | 63.36 | 1.03 | 0.3043 |
| **Congestive heart failure** |  | 110.57 | 58.34 | 1.90 | 0.0581 |
| **Hypertension** |  | 55.53 | 93.51 | 0.59 | 0.5526 |
| **Dialysis** |  | 1297.62 | 183.83 | 7.06 | 0.0000*** |
|  |  | **edf** | **Ref.df** | **F** | **p-value** |
| **s(PDC mean ACE inhibitors) male** |  | 1.00 | 1.00 | 2.95 | 0.0862 |
| **s(PDC mean ACE inhibitors) female** |  | 4.79 | 4.79 | 3.68 | 0.0047** |
| **s(PDC mean β-blockers) male** |  | 1.00 | 1.00 | 3.90 | 0.0484* |
| **s(PDC mean β-blockers) female** |  | 3.78 | 3.78 | 1.52 | 0.2812 |
| **s(PDC mean statins) male** |  | 1.20 | 1.20 | 0.79 | 0.3153 |
| **s(PDC mean statins) female** |  | 5.18 | 5.18 | 4.88 | 0.0001*** |
| **s(PDC mean anti-platelet agents) male** |  | 1.00 | 1.00 | 34.63 | 0.0000*** |
| **s(PDC mean anti-platelet agents) female** |  | 1.78 | 1.78 | 10.23 | 0.0001*** |
| **s(PDC standard deviation ACE inhibitors) male** |  | 1.00 | 1.00 | 0.00 | 0.9596 |
| **s(PDC standard deviation ACE inhibitors) female** |  | 6.75 | 6.75 | 9.03 | 0.0000*** |
| **s(PDC standard deviation β-blockers) male** |  | 2.86 | 2.86 | 12.70 | 0.0000*** |
| **s(PDC standard deviation β-blockers) female** |  | 1.78 | 1.78 | 4.62 | 0.0365* |
| **s(PDC standard deviation statins) male** |  | 1.00 | 1.00 | 0.10 | 0.7508 |
| **s(PDC standard deviation statins) female** |  | 7.72 | 7.72 | 7.28 | 0.0000*** |
| **s(PDC standard deviation anti-platelet agents) male** |  | 1.00 | 1.00 | 14.30 | 0.0002*** |
| **s(PDC standard deviation anti-platelet agents) female** |  | 1.00 | 1.00 | 4.61 | 0.0318* |
| R-sq. (adj.) = 0.109 | | | | | |
| **Abbreviations: AMI (Acute Myocardial infarction), BIMD 2010 (Bavarian Index of Multiple Deprivation, year 2010), BMI (Body Mass Index), DMP (Disease Management Program), HMG (Hierarchical Morbidity Group), NYHA (New York Hear Association), PDC (Proportion of days covered)** | | | | | |

Online table 3: Base Case - Influence of PDC rates on hospitalization costs

| **N = 9,287** |  | **Estimate** | **Std. Error** | **t value** | **Pr(>\|t\|)** |
| --- | --- | --- | --- | --- | --- |
| **(Intercept)** |  | 1026.21 | 1691.08 | 0.61 | 0.5440 |
| **Age** | **55 < 65** | 745.77 | 613.65 | 1.22 | 0.2243 |
|  | **65 < 75** | 334.04 | 572.80 | 0.58 | 0.5598 |
|  | **≥ 75** | -1272.72 | 577.78 | -2.20 | 0.0276* |
| **Gender** | **female** | -539.48 | 276.40 | -1.95 | 0.0510 |
| **BMI** | **underweight** | 974.71 | 1612.91 | 0.60 | 0.5456 |
|  | **overweight** | -317.95 | 324.81 | -0.98 | 0.3277 |
|  | **obese** | -416.86 | 352.50 | -1.18 | 0.2370 |
| **BIMD 2010 (Q1 least deprived,Q5 most deprived)** | **Q2** | -717.41 | 381.89 | -1.88 | 0.0603 |
|  | **Q3** | -1265.17 | 410.00 | -3.09 | 0.0020** |
|  | **Q4** | -408.55 | 405.22 | -1.01 | 0.3134 |
|  | **Q5** | -769.68 | 384.49 | -2.00 | 0.0453* |
| **Smoker** | **yes** | 379.75 | 398.67 | 0.95 | 0.3408 |
| **NYHA** | **1** | 2354.24 | 721.75 | 3.26 | 0.0011** |
|  | **2** | 1542.18 | 429.01 | 3.59 | 0.0003*** |
|  | **3** | 2752.95 | 386.56 | 7.12 | 0.0000*** |
|  | **4** | 4239.42 | 406.65 | 10.43 | 0.0000*** |
| **DMP COPD** | **yes** | 502.34 | 460.95 | 1.09 | 0.2758 |
| **DMP asthma** | **yes** | -1071.36 | 778.48 | -1.38 | 0.1688 |
| **DMP diabetes type 1** | **yes** | -2289.53 | 2170.80 | -1.05 | 0.2916 |
| **DMP diabetes type 2** | **yes** | 203.09 | 256.90 | 0.79 | 0.4292 |
| **deceased** | **yes** | 10163.05 | 817.01 | 12.44 | 0.0000*** |
| **HMG assignments per month** |  | 0.06 | 0.22 | 0.28 | 0.7826 |
| **Year after AMI** |  | -5133.15 | 147.99 | -34.69 | 0.0000*** |
| **days insured** |  | 23.29 | 4.15 | 5.60 | 0.0000*** |
| **Angina pectoris** |  | 2108.33 | 248.96 | 8.47 | 0.0000*** |
| **Peripheral vascular disease** |  | 3924.25 | 350.33 | 11.20 | 0.0000*** |
| **Dyslipidemia** |  | 396.41 | 323.76 | 1.22 | 0.2208 |
| **Congestive heart failure** |  | 1922.45 | 312.64 | 6.15 | 0.0000*** |
| **Hypertension** |  | 1675.99 | 508.58 | 3.30 | 0.0010*** |
| **Dialysis** |  | 6594.20 | 879.18 | 7.50 | 0.0000*** |
|  |  | **edf** | **Ref.df** | **F** | **p-value** |
| **s(PDC mean ACE inhibitors) male** |  | 2.03 | 2.03 | 1.85 | 0.1693 |
| **s(PDC mean ACE inhibitors) female** |  | 1.00 | 1.00 | 0.07 | 0.7915 |
| **s(PDC mean β-blockers) male** |  | 1.00 | 1.00 | 2.64 | 0.1042 |
| **s(PDC mean β-blockers) female** |  | 1.00 | 1.00 | 0.17 | 0.6823 |
| **s(PDC mean statins) male** |  | 1.52 | 1.52 | 1.42 | 0.1475 |
| **s(PDC mean statins) female** |  | 1.00 | 1.00 | 1.23 | 0.2682 |
| **s(PDC mean anti-platelet agents) male** |  | 3.16 | 3.16 | 17.17 | 0.0000*** |
| **s(PDC mean anti-platelet agents) female** |  | 1.00 | 1.00 | 2.36 | 0.1242 |
| **s(PDC standard deviation ACE inhibitors) male** |  | 1.17 | 1.17 | 0.08 | 0.8695 |
| **s(PDC standard deviation ACE inhibitors) female** |  | 1.73 | 1.73 | 3.94 | 0.0146* |
| **s(PDC standard deviation β-blockers) male** |  | 3.29 | 3.29 | 9.89 | 0.0000*** |
| **s(PDC standard deviation β-blockers) female** |  | 1.00 | 1.00 | 0.71 | 0.4004 |
| **s(PDC standard deviation statins) male** |  | 1.00 | 1.00 | 0.22 | 0.6423 |
| **s(PDC standard deviation statins) female** |  | 2.82 | 2.82 | 1.88 | 0.2541 |
| **s(PDC standard deviation anti-platelet agents) male** |  | 4.42 | 4.42 | 4.18 | 0.0021** |
| **s(PDC standard deviation anti-platelet agents) female** |  | 2.39 | 2.39 | 3.32 | 0.0288* |
| R-sq. (adj.) = 0.238 | | | | | |
| **Abbreviations: AMI (Acute Myocardial infarction), BIMD 2010 (Bavarian Index of Multiple Deprivation, year 2010), BMI (Body Mass Index), DMP (Disease Management Program), HMG (Hierarchical Morbidity Group), NYHA (New York Hear Association), PDC (Proportion of days covered)** | | | | | |

Online table 4: Base Case – Influence of PDC rates on rehabilitation costs

| **N = 9,287** |  | **Estimate** | **Std. Error** | **t value** | **Pr(>\|t\|)** |
| --- | --- | --- | --- | --- | --- |
| **(Intercept)** |  | 321.62 | 186.23 | 1.73 | 0.0842 |
| **Age** | **55 < 65** | 185.75 | 65.20 | 2.85 | 0.0044** |
|  | **65 < 75** | 384.60 | 60.68 | 6.34 | 0.0000*** |
|  | **≥ 75** | 313.01 | 61.23 | 5.11 | 0.0000*** |
| **Gender** | **female** | 116.21 | 29.28 | 3.97 | 0.0001*** |
| **BMI** | **underweight** | -23.84 | 173.27 | -0.14 | 0.8906 |
|  | **overweight** | -19.48 | 34.79 | -0.56 | 0.5755 |
|  | **obese** | -33.52 | 37.57 | -0.89 | 0.3723 |
| **BIMD 2010 (Q1 least deprived,Q5 most deprived)** | **Q2** | -15.82 | 40.35 | -0.39 | 0.6949 |
|  | **Q3** | 9.07 | 43.35 | 0.21 | 0.8343 |
|  | **Q4** | -86.18 | 42.86 | -2.01 | 0.0444* |
|  | **Q5** | -73.04 | 40.62 | -1.80 | 0.0722 |
| **Smoker** | **yes** | -62.01 | 42.48 | -1.46 | 0.1444 |
| **NYHA** | **1** | -3.50 | 76.95 | -0.05 | 0.9637 |
|  | **2** | 74.10 | 45.94 | 1.61 | 0.1068 |
|  | **3** | 114.47 | 41.47 | 2.76 | 0.0058** |
|  | **4** | 250.81 | 43.68 | 5.74 | 0.0000*** |
| **DMP COPD** | **yes** | 89.35 | 49.11 | 1.82 | 0.0689 |
| **DMP asthma** | **yes** | -114.13 | 82.42 | -1.38 | 0.1662 |
| **DMP diabetes type 1** | **yes** | -146.78 | 230.56 | -0.64 | 0.5244 |
| **DMP diabetes type 2** | **yes** | 15.21 | 27.28 | 0.56 | 0.5770 |
| **deceased** | **yes** | 17.68 | 90.52 | 0.20 | 0.8452 |
| **HMG assignments per month** |  | -0.10 | 0.02 | -4.12 | 0.0000*** |
| **Year After AMI** |  | -381.29 | 16.78 | -22.72 | 0.0000*** |
| **days insured** |  | 0.44 | 0.46 | 0.95 | 0.3411 |
| **Angina pectoris** |  | 57.48 | 27.05 | 2.12 | 0.0336* |
| **Peripheral vascular disease** |  | 263.59 | 38.63 | 6.82 | 0.0000*** |
| **Dyslipidemia** |  | 11.47 | 35.20 | 0.33 | 0.7444 |
| **Congestive heart failure** |  | 67.65 | 34.17 | 1.98 | 0.0478* |
| **Hypertension** |  | 97.77 | 55.72 | 1.75 | 0.0793 |
| **Dialysis** |  | 141.90 | 95.61 | 1.48 | 0.1378 |
|  |  | **edf** | **Ref.df** | **F** | **p-value** |
| **s(PDC mean ACE inhibitors) male** |  | 1.00 | 1.00 | 0.09 | 0.7605 |
| **s(PDC mean ACE inhibitors) female** |  | 1.00 | 1.00 | 1.01 | 0.3147 |
| **s(PDC mean β-blockers) male** |  | 1.00 | 1.00 | 1.61 | 0.2046 |
| **s(PDC mean β-blockers) female** |  | 1.00 | 1.00 | 0.36 | 0.5478 |
| **s(PDC mean statins) male** |  | 1.00 | 1.00 | 6.18 | 0.0129* |
| **s(PDC mean statins) female** |  | 1.61 | 1.61 | 3.03 | 0.0503 |
| **s(PDC mean anti-platelet agents) male** |  | 1.00 | 1.00 | 2.56 | 0.1096 |
| **s(PDC mean anti-platelet agents) female** |  | 1.00 | 1.00 | 1.64 | 0.2009 |
| **s(PDC standard deviation ACE inhibitors) male** |  | 1.95 | 1.95 | 1.28 | 0.2377 |
| **s(PDC standard deviation ACE inhibitors) female** |  | 1.96 | 1.96 | 4.76 | 0.0060* |
| **s(PDC standard deviation β-blockers) male** |  | 1.67 | 1.67 | 0.77 | 0.3046 |
| **s(PDC standard deviation β-blockers) female** |  | 1.00 | 1.00 | 0.31 | 0.5750 |
| **s(PDC standard deviation statins) male** |  | 1.00 | 1.00 | 0.08 | 0.7809 |
| **s(PDC standard deviation statins) female** |  | 2.77 | 2.77 | 2.34 | 0.1042 |
| **s(PDC standard deviation anti-platelet agents) male** |  | 2.54 | 2.54 | 2.78 | 0.0344* |
| **s(PDC standard deviation anti-platelet agents) female** |  | 3.08 | 3.08 | 4.67 | 0.0029** |
| R-sq. (adj.) = 0.0997 | | | | | |
| **Abbreviations: AMI (Acute Myocardial infarction), BIMD 2010 (Bavarian Index of Multiple Deprivation, year 2010), BMI (Body Mass Index), DMP (Disease Management Program), HMG (Hierarchical Morbidity Group), NYHA (New York Hear Association), PDC (Proportion of days covered)** | | | | | |

Online table 5: Base Case – Influence of PDC rates on remedy and aid costs

| **N = 9,287** |  | **Estimate** | **Std. Error** | **t value** | **Pr(>\|t\|)** |
| --- | --- | --- | --- | --- | --- |
| **(Intercept)** |  | -701.03 | 171.48 | -4.09 | 0.0000*** |
| **Age** | **55 < 65** | -30.39 | 70.66 | -0.43 | 0.6672 |
|  | **65 < 75** | -55.84 | 67.07 | -0.83 | 0.4051 |
|  | **≥ 75** | -41.28 | 67.62 | -0.61 | 0.5415 |
| **Gender** | **female** | 31.33 | 33.01 | 0.95 | 0.3426 |
| **BMI** | **underweight** | -36.01 | 179.37 | -0.20 | 0.8409 |
|  | **overweight** | -31.75 | 36.27 | -0.88 | 0.3813 |
|  | **obese** | 13.87 | 40.32 | 0.34 | 0.7309 |
| **BIMD 2010 (Q1 least deprived,Q5 most deprived)** | **Q2** | -29.24 | 45.83 | -0.64 | 0.5235 |
|  | **Q3** | 35.49 | 48.96 | 0.72 | 0.4685 |
|  | **Q4** | -3.67 | 48.49 | -0.08 | 0.9397 |
|  | **Q5** | -5.38 | 45.99 | -0.12 | 0.9068 |
| **Smoker** | **yes** | -107.12 | 45.53 | -2.35 | 0.0187* |
| **NYHA** | **1** | 130.84 | 82.61 | 1.58 | 0.1133 |
|  | **2** | 88.27 | 48.13 | 1.83 | 0.0667 |
|  | **3** | 150.31 | 42.95 | 3.50 | 0.0005*** |
|  | **4** | 217.59 | 45.02 | 4.83 | 0.0000*** |
| **DMP COPD** | **yes** | 178.09 | 53.03 | 3.36 | 0.0008*** |
| **DMP asthma** | **yes** | 189.08 | 92.37 | 2.05 | 0.0407* |
| **DMP diabetes type 1** | **yes** | 71.70 | 251.69 | 0.28 | 0.7758 |
| **DMP diabetes type 2** | **yes** | 60.90 | 29.96 | 2.03 | 0.0421* |
| **deceased** | **yes** | 370.07 | 80.36 | 4.60 | 0.0000*** |
| **HMG assignments per month** |  | 0.15 | 0.02 | 6.97 | 0.0000*** |
| **Year after AMI** |  | -35.16 | 13.17 | -2.67 | 0.0076** |
| **days insured** |  | 2.38 | 0.41 | 5.79 | 0.0000*** |
| **Angina pectoris** |  | -21.23 | 25.72 | -0.83 | 0.4091 |
| **Peripheral vascular disease** |  | 3.52 | 34.13 | 0.10 | 0.9180 |
| **Dyslipidemia** |  | 1.33 | 33.50 | 0.04 | 0.9683 |
| **Congestive heart failure** |  | 47.92 | 31.63 | 1.52 | 0.1298 |
| **Hypertension** |  | 149.02 | 51.02 | 2.92 | 0.0035** |
| **Dialysis** |  | -136.93 | 92.53 | -1.48 | 0.1390 |
|  |  | **edf** | **Ref.df** | **F** | **p-value** |
| **s(PDC mean ACE inhibitors) male** |  | 1.00 | 1.00 | 0.41 | 0.5228 |
| **s(PDC mean ACE inhibitors) female** |  | 1.74 | 1.74 | 1.44 | 0.1482 |
| **s(PDC mean β-blockers) male** |  | 1.00 | 1.00 | 0.43 | 0.5110 |
| **s(PDC mean β-blockers) female** |  | 1.00 | 1.00 | 0.07 | 0.7850 |
| **s(PDC mean statins) male** |  | 2.08 | 2.08 | 3.36 | 0.0353* |
| **s(PDC mean statins) female** |  | 1.00 | 1.00 | 0.19 | 0.6613 |
| **s(PDC mean anti-platelet agents) male** |  | 1.00 | 1.00 | 6.60 | 0.0102* |
| **s(PDC mean anti-platelet agents) female** |  | 2.60 | 2.60 | 1.76 | 0.2405 |
| **s(PDC standard deviation ACE inhibitors) male** |  | 1.00 | 1.00 | 0.07 | 0.7879 |
| **s(PDC standard deviation ACE inhibitors) female** |  | 1.12 | 1.12 | 4.62 | 0.0235* |
| **s(PDC standard deviation β-blockers) male** |  | 4.19 | 4.19 | 6.84 | 0.0000*** |
| **s(PDC standard deviation β-blockers) female** |  | 2.70 | 2.70 | 8.83 | 0.0001*** |
| **s(PDC standard deviation statins) male** |  | 1.00 | 1.00 | 0.77 | 0.3804 |
| **s(PDC standard deviation statins) female** |  | 1.44 | 1.44 | 1.82 | 0.2660 |
| **s(PDC standard deviation anti-platelet agents) male** |  | 1.00 | 1.00 | 3.34 | 0.0675 |
| **s(PDC standard deviation anti-platelet agents) female** |  | 1.05 | 1.05 | 5.69 | 0.0149* |
| R-sq. (adj.) = 0.0417 | | | | | |
| **Abbreviations: AMI (Acute Myocardial infarction), BIMD 2010 (Bavarian Index of Multiple Deprivation, year 2010), BMI (Body Mass Index), DMP (Disease Management Program), HMG (Hierarchical Morbidity Group), NYHA (New York Hear Association), PDC (Proportion of days covered)** | | | | | |
